# Supplementary material for: Self-assembly using a retro Diels-Alder reaction
Source: Nat Commun. 2021 Jul 9;12:4207. doi: 10.1038/s41467-021-24492-z (PMC8270933; doi:10.1038/s41467-021-24492-z)
Supplement: Supplementary file 1 — Supplementary Information [file 41467_2021_24492_MOESM1_ESM.pdf]

Supplementary Information

Self-assembly using a retro Diels-Alder reaction

Jaeyoung Park,[1, †] Jung-Moo Heo,[1, †] Sicheon Seong,[2] Jaegeun Noh[2,3] and Jong-Man Kim[1,3 *]

[1]Department of Chemical Engineering, Hanyang University, Seoul 04763, Korea.

[2]Department of Chemistry, Hanyang University, Seoul 04763, Korea

[3]Institute of Nano Science and Technology, Hanyang University, Seoul 04763, Korea

*E-mail: jmk@hanyang.ac.kr (J.-M. Kim)

[†] These authors contributed equally to this work.

Contents **Page no.**

1. Supplementary Methods

Instruments……………………………………………………………….............2

Materials………………………………………………………………….............2

Synthesis of Furan and Maleimide Substituted Diacetylenes F and M………….3

Synthesis of 1-exo and 1-endo…………………………………………….……….5

Retro Diels-Alder Reaction, Self-Assembly, Polymerization of 1-endo.................8

Cysteine-Promoted Color Change of Polydiacetylene (PDA)................................9

Thermochromic Reversibility Test...10

^{1}H, ^{13}C NMR and mass Spectra for F, M, 1-exo and 1-endo…,……..…………11

Supplementary Figures 7-25……………,…………………………….……......19

Supplementary Table 1…..……,……………………………………...............38

2. Supplementary References..38

1. Supplementary Methods

Instruments

Raman spectra were recorded on a LabRAM HR Evolution Raman spectrometer (Horiba Scientific, 785 nm laser source). The UV absorption spectra and transmittance spectra were recorded on a single beam Agilent 8453 UV−Vis spectrometer (Agilent Technologies, Waldbronn, Germany). IR spectra were recorded on a Thermo Nicolet NEXUS 470 FTIR uisng an ATR accessory (Thermo Fisher Scientific, Inc.). The ^{1}H and ^{13}C NMR spectra were recorded on a Varian Unitylnova (600 MHz) spectrometer at 298 K in $CDCl_3$. High-resolution mass spectra (HRMS) were recorded on a SYNAPT G2 (water, U.K.) using a time-of-flight (TOF) analyzer.

Materials

The intermediates Diels-Alder adducts, DA-exo and DA-endo, were synthesized according to the literature procedures.[1] 10,12-Pentacosadiynoic acid (PCDA) was obtained from GFS Chemicals (Powell, OH). Furfuryl alcohol and N-(4-Hydroxy phenyl) maleimide were purchased from Sigma Aldrich. All chemicals were used as received without purification.

Synthesis of Furan and Maleimide Substituted Diacetylenes F and M

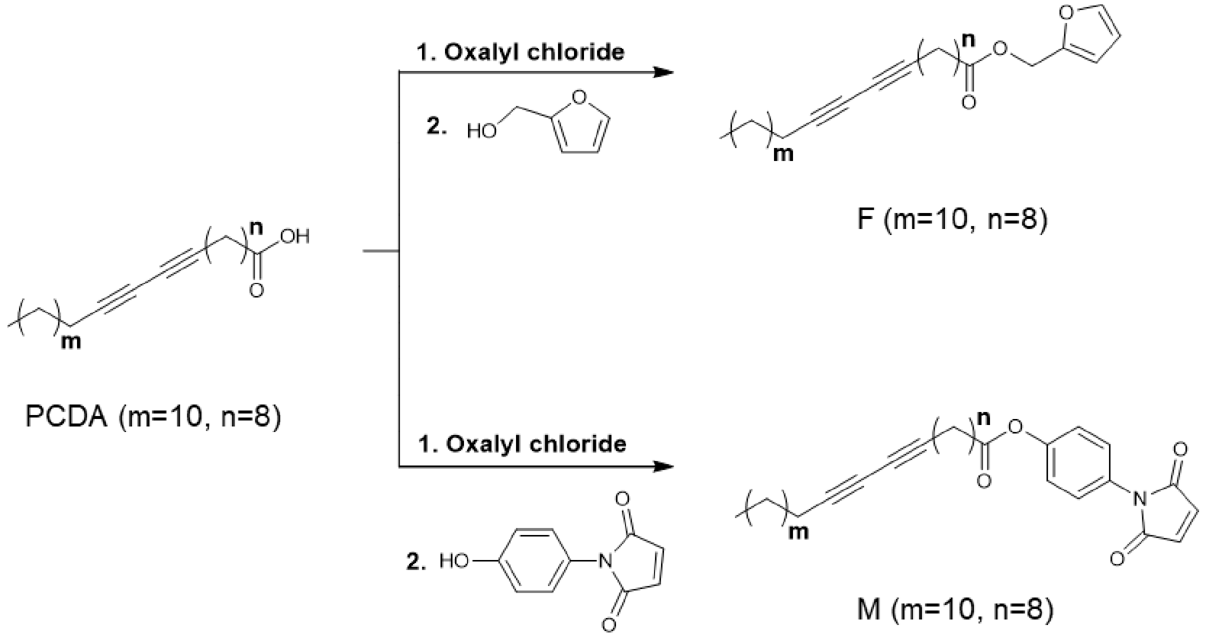

Supplementary Figure 1. Synthetic routes for F and M.

Synthesis of F.

To a solution of 10,12-pentacosadiynoic acid (PCDA, 2.0 g, 5.34 mmol) in dichloromethane (15 mL) was added oxalyl chloride (1.3 g, 10.68 mmol) followed by a drop of DMF at 0 °C under argon atmosphere. The resulting solution was stirred at ambient temperature for 6 h and evaporated to dryness under reduced pressure. The residual powder was redissolved in dichloromethane (10 mL) and was added to the solution of furfuryl alcohol (0.64 g, 6.54 mmol) and triethylamine (1.5 mL, 10.68 mmol) in dichloromethane (10 mL). The resultant mixture was stirred at room temperature for 15 h, concentrated *in vacuo* and the residue was purified by a silica gel column chromatography (hexane/ethyl acetate, 80/20 vol%) to yield the desired product F (1.8 g, 60%). m.p: 38-40 ºC; ^{1}H NMR (600 MHz, $CDCl_3$): 7.42 (dd, J_1 = 0.6 Hz, J_2 = 1.8 Hz, 1H), 6.40 (d, J = 3.0 Hz, 1H), 6.36 (t, J = 2.4 Hz, 1H), 5.06 (s, 2H), 2.32 (t, J = 7.8 Hz, 2H), 2.23 (t, J = 7.2 Hz, 4H), 1.63~1.48 (m, 8H), 1.38~1.25 (m, 26H), 0.88 (t, J = 6.6 Hz, 3H); ^{13}C NMR (150 MHz, $CDCl_3$): δ 173.4, 149.6, 143.2, 110.5, 110.4, 77.6, 77.4, 57.8, 34.1,

31.9, 29.7, 29.6, 29.5, 29.4, 29.3, 29.1, 29.0, 28.9, 28.8, 28.7, 28.6, 28.4, 28.3, 24.8, 22.7, 19.2, 19.1, 14.1; IR (ATR) vcm^{-1}:3452, 3128, 2958, 2916, 2846, 2177, 2141, 1732, 1620, 1568, 1504, 1460, 1423, 1381, 1344, 1319, 1308, 1286, 1250, 1227, 1207, 1171, 1097, 1078, 1047, 1016, 985, 953, 914, 885, 822, 742, 725, 600; MS (MALDI-TOF, *m/z*): exact mass calculated for $C_{30}H_{46}O_3$ required 454.34 found 454.04.

Synthesis of M.

To a solution of 10,12-pentacosadiynoic acid (2.0 g, 5.34 mmol) in dichloromethane (15 mL) was added oxalyl chloride (1.3 g, 10.68 mmol) followed by a drop of DMF at 0 °C under argon atmosphere. The resulting solution was stirred at ambient temperature for 6 h and evaporated to dryness under reduced pressure. The residual powder was redissolved in dichloromethane (10 mL) and was added to the solution of N-(4-hydroxy phenyl) maleimide (1.2 g, 6.54 mmol) and triethylamine (1.5 mL, 10.68 mmol) in dichloromethane (10 mL). The reaction mixture was stirred at room temperature for 15 h, evaporated to dryness and the residue was purified by a silica gel column chromatography (hexane/ethyl acetate, 80/20 vol%) to yield the desired product M (0.4 g, 13%) as a yellow solid. m.p: 71-73 °C; ^{1}H NMR (600 MHz, $CDCl_3$): 7.37 (d, J = 9.0 Hz, 2H), 7.19 (d, J = 9.0 Hz, 2H), 6.86 (s, 2H), 2.57 (t, J = 7.2 Hz, 2H), 2.24 (q, J = 7.2 Hz, 4H), 1.75 (quint., J = 7.8 Hz, 2H), 1.51 (quint., J = 7.8 Hz, 4H), 1.42~1.25 (m, 26H), 0.88 (t, J = 6.6 Hz, 3H); ^{13}C NMR (150 MHz, $CDCl_3$): δ 171.9, 169.3, 149.9, 134.2, 128.5, 126.9, 123.0, 77.6, 77.4, 65.2, 34.3, 31.9, 29.8, 29.7, 29.6, 29.5, 29.3, 29.2, 29.1, 29.0, 28.9, 28.8, 28.7, 28.4, 28.3, 24.8, 22.7, 19.3, 19.2, 14.1; IR (ATR) vcm^{-1}: 3099, 3074, 2952, 2920, 2848, 1759, 1705, 1601, 1512, 1464, 1408, 1396, 1377, 1288, 1246, 1213, 1196, 1157, 1138, 1093, 1038, 1016, 951, 926, 864, 835, 723, 710, 688; MS (MALDI-TOF, *m/z*): exact mass calculated for $C_{35}H_{49}NO_4$ required 547.37 found 547.14.

Synthesis of 1-exo and 1-endo.

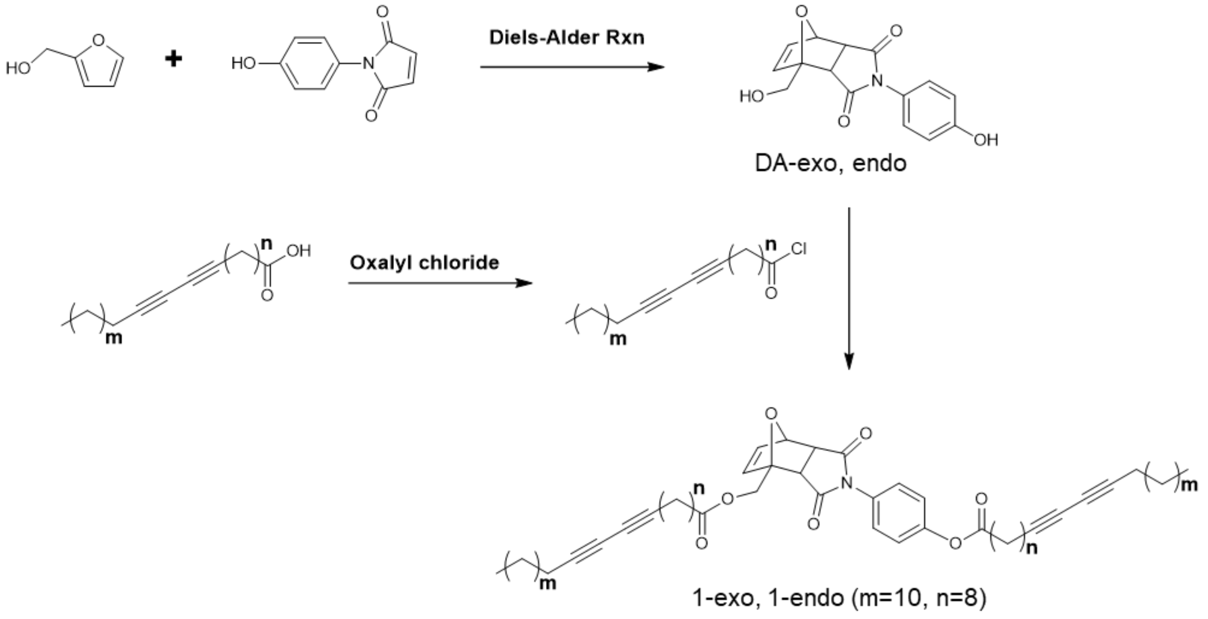

Supplementary Figure 2. Synthetic routes for 1-exo and 1-endo**.**

Synthesis of intermediate Diels-Alder adduct DA-exo.

To a solution of N-(4-hydroxyphenyl)maleimide (220 mg, 2.04 mmol) in acetone (8.0 mL) was added fufuryl alcohol (400 mg, 2.04 mmol). The reaction was allowed to stir at 55 °C until TLC showed consumption of the starting materials. The reaction mixture was concentrated to dryness and the residue was purified by a flash silica gel chromatography (dichloromethane/methanol, 90/10 vol%) to yield the desired product DA-exo (440 mg, 71%). ^{1}H NMR (600 MHz, DMSO-d_6): 9.74 (s, 1H), 6.95 (d, J = 8.4 Hz, 2H), 6.82 (d, J = 9.0 Hz, 2H), 6.56~6.54 (m, 2H), 5.16 (s, 1H), 4.99 (t, J = 6.0 Hz, 1H), 4.07 (dd, J_1 = 6.0 Hz, J_2 = 12.6 Hz, 1H), 3.75 (dd, J_1 = 5.4 Hz, J_2 = 12.6 Hz,), 3.13 (d, J = 6.6 Hz, 1H), 2.96 (d, J = 6.6 Hz, 1H). The purified DA-exo was found to be unstable and was used for the synthesis of 1-exo without further spectral analyses.

Synthesis of 1-exo.

To a solution of 10,12-pentacosadiynoic acid (200 mg, 0.53 mmol, 2.4 Equiv.) in dichloromethane (5 mL) was added oxalyl chloride (135 mg, 1.07 mmol) followed by a drop of DMF at 0 °C under nitrogen atmosphere. The resulting solution was stirred at ambient temperature for 6 h and evaporated to dryness under reduced pressure. The residue was dissolved in dichloromethane (7 mL) and the resultant solution was added to the solution of DA-exo (80 mg, 0.22 mmol) and triethylamine (110 mg, 1.07 mmol) in dichloromethane (7 mL). The reaction mixture was stirred at room temperature for 15 h, evaporated to dryness and the residue was purified by a silica gel column chromatography to afford the desired 1-exo as a white solid (100 mg, 40%). m.p: 50-52 °C; ^{1}H NMR (600 MHz, $CDCl_3$): 7.30 (d, J = 8.4 Hz, 2H), 7.19 (d, J = 9.0 Hz, 2H), 6.62 (d, J = 5.4 Hz, 1H), 6.47 (d, J = 5.4 Hz, 1H), 5.40 (d, J = 1.2 Hz, 1H), 5.02 (d, J = 9.6 Hz, 1H), 4.49 (d, J = 9.6 Hz, 1H), 3.15 (d, J = 6.6 Hz, 1H), 3.05 (d, J = 6.6 Hz, 1H), 2.56 (t, J = 7.2 Hz, 2H), 2.35 (t, J = 7.8 Hz, 2H), 2.26~2.22 (m, 8H), 1.74 (quint, J = 7.8 Hz, 2H), 1.64~1.25 (m, 64H), 0.88 (t, J = 6.6 Hz, 6H); ^{13}C NMR (150 MHz, $CDCl_3$): δ 174.6, 173.2, 173.1, 171.7, 150.6, 137.6, 137.3, 127.5, 122.3, 90.0, 81.5, 77.4, 65.2, 61.1, 50.0, 48.4, 34.3, 34.0, 31.9, 29.8, 29.7, 29.6, 29.5, 29.3, 29.2, 29.1, 29.0, 28.9, 28.8, 28.7, 28.6, 28.5, 28.4, 28.3, 28.2, 28.1, 24.9, 24.8, 22.7, 19.3, 19.2, 19.1, 14.1; IR (ATR) vcm^{-1}: 2922, 2850, 1753, 1736, 1714, 1703, 1508, 1468, 1419, 1390, 1321, 1286, 1248, 1207, 1165, 1142, 1101, 1076, 1016, 982, 922, 889, 847, 808, 719, 669, 644, 598, 526; HRMS (ESI, *m/z*): exact mass calculated for $C_{65}H_{93}NO_7Na$ required 1022.6850 found 1022.6851.

Synthesis of intermediate Diels-Alder adduct DA-endo.

N-(p-hydroxyphenyl)-maleimide (500 mg, 2.64 mmol) and fufuryl alcohol (310 mg, 3.17 mmol) were dissolved in anhydrous acetonitrile (4.0 mL) under a nitrogen atmosphere in a flame-dried flask equipped with a magnetic stirring-bar. The reaction mixture was stirred at

35 °C for 18 h, concentrated under reduced pressure. A silica gel column column chromatography (6:4 to 4:6, hexane:ethyl acetate) afforded a mixture of DA-exo and DA-endo (1 : 4). ^{1}H NMR (600 MHz, DMSO-d_6): 9.72 (s, 1H), 6.89 (d, J = 9.4 Hz, 2H), 6.79 (d, J = 9.4 Hz, 2H), 6.58~6.55 (m, 2H), 5.32~5.31 (m, 1H), 3.74 (t, J = 5.4 Hz, 1H), 3.52 (d, J = 6.0 Hz, 1H), 3.15 (d, J = 6.0 Hz, 1H), 2.98 (d, J = 6.0 Hz, 1H). The mixture was used for the preparation of 1-endo without further purification.

Synthesis of 1-endo.

To a solution of 10,12-pentacosadiynoic acid (200 mg, 0.53 mmol) in dichloromethane (5 mL) was added oxalyl chloride (135 mg, 1.07 mmol) followed by a drop of DMF at 0 °C under nitrogen atmosphere. The resulting solution was stirred at ambient temperature for 6 h and evaporated to dryness under reduced pressure. The residue was dissolved in dichloromethane (7 mL) and the solution was added to the solution of DA-endo (contains ca. 25% of DA-exo as described above) (80 mg, 0.22 mmol) and triethylamine (110 mg, 1.07 mmol) in dichloromethane (7 mL). The reaction mixture was stirred at room temperature for 15 h. The reaction mixture was evaporated to dryness and the residue was purified by a silica gel column chromatography to afford the desired 1-endo as a white solid (90 mg, 38%). m.p: 67-69 °C; ^{1}H NMR (600 MHz, $CDCl_3$): 7.17~7.14 (m, 4H), 6.60 (d, J = 6.0 Hz, 1H), 6.46 (d, J = 6.0 Hz, 1H), 5.42 (d, J = 5.4 Hz, 1H), 4.91 (d, J = 9.6 Hz, 1H), 4.65 (d, J = 9.0 Hz, 1H), 3.83~3.81 (m, 1H), 3.55 (d, J = 8.4 Hz, 1H), 2.55 (t, J = 7.2 Hz, 2H), 2.39 (t, J = 7.8 Hz, 2H), 2.26~2.22 (m, 8H), 1.73 (quint, J = 7.2 Hz, 2H), 1.64 (quint, J = 7.2 Hz, 2H), 1.53~1.48 (m, 8H), 1.39~1.25 (m, 52H), 0.88 (t, J = 6.6 Hz, 6H); ^{13}C NMR (150 MHz, $CDCl_3$): δ 173.4, 173.2, 173.1, 171.7, 150.6, 135.8, 134.7, 128.6, 127.2, 122.3, 90.1, 79.9, 77.6, 77.5, 77.4, 65.3, 65.2, 61.7, 47.6, 46.6, 34.3, 33.9, 31.9, 29.8, 29.7, 29.6, 29.5, 29.3, 29.2, 29.1, 29.0, 28.9, 28.8, 28.7, 28.6, 28.5, 28.4, 28.3, 24.8, 24.7, 22.7, 19.3, 19.2, 14.1; IR (ATR) νcm^{-1}: 2920, 2850, 1763, 1732, 1705,

1508, 1469, 1417, 1400, 1379, 1325, 1282, 1249, 1209, 1188, 1173, 1161, 1122, 1099, 1076, 1032, 1018, 991, 972, 955, 945, 920, 881, 858, 802, 764, 746, 721, 700, 671, 640, 629, 525; HRMS (ESI, *m/z*): exact mass calculated for $C_{65}H_{93}NO_7Na$ required 1022.6850 found 1022.6845.

Retro Diels-Alder Reaction, Self-Assembly, Polymerization of 1-endo

Glass substrate

The powder form of 1-endo (3 mg) was placed on a clean glass substrate and the glass was heated at 110 °C for 1 min on a hot plate. The heat-treated glass was placed in a freezer (-10 °C) for 10 min to stabilization and induce self-assembly of the retro Diels-Alder products. Photopolymerization was conducted by irradiating with a common hand-held 254 nm laboratory UV lamp (1 mW/cm^2) for 1 min. Appearance of an intense blue color was observed by the UV irradiation, confirming the PDA formation.

Analysis of the retro Diels-Alder products displayed in Fig. 3. was carried out by dissolving the heat-treated (110 °C, 1 min and -10 °C, 10 min) sample in $CDCl_3$ and the product distribution was calculated by ^{1}H NMR analysis. By employing a similar method, temperature (Fig. 4) and time (Fig. 5)-dependent product distribution was measured.

Filter paper

The compound 1-endo (10 mg) was dissolved in 1 mL of ethyl acetate (final concentration: 10 mM) and 100 µL of the resulting solution was drop-casted on a filter paper (diameter: 2 cm). The 1-endo immobilized filter paper was placed on a hot plate (110 °C) for 5 min to induce the retro Diels-Alder reaction. UV irradiation (254 nm, 1 mW/cm^2, 1 min) to the filter paper afforded generation of a blue-colored PDA. The degree of polymerization was dependent on

the stabilization time and the absorption at 620 nm (PDA) was increased when the filter paper was kept at 25 °C for 20 min or at -10 °C (freezer) for 10 min (Supplementary Fig. 8).

In order to calculate the conversion of retro Diels-Alder products, F and M to PDA, 60 mg of 1-endo was dissolved in ethyl acetate (1 mL) and the resulting solution was drop-casted on two different filter papers (diameter: 5.5 cm), 500 µL each. One of them was only heat-treated (110 °C, 5 min and -10 °C, 10 min), and another one was UV irradiated (254 nm, 1 mW/cm^2, 1 min) after heat treatment for photopolymerization of F and M. The filter papers were washed using 50 mL of ethyl acetate. Since the polymer is insoluble in ethyl acetate and the residual F, M and 1-endo are soluble in this solvent, washing with ethyl acetate allows effective isolation of the unpolymerized F, M and 1-endo. The solution was concentrated and the residue was dissolved in $CDCl_3$ and analyzed by using ^{1}H NMR spectroscopy to calculate amount of unpolymerized F and M (see Supplementary Fig. 25 and Supplementary Table 1).

Patterned PDA images shown in Supplementary Fig. 12 was obtained by placing patterned hot metal wires on the 1-endo immobilized filter paper (diameter: 5.5 cm) followed by UV irradiated (254 nm, 1 mW/cm^2, 1 min) after removing of the metal wires.

Cysteine-Promoted Color Change of Polydiacetylene (PDA)

A 1-endo immobilized filter paper was placed on a hot plate (110 °C) for 5 min to induce the retro Diels-Alder reaction and stabilized in a freezer (-10 °C) for 10 min. UV irradiation (254 nm, 1 mW/cm^2, 1 min) to the filter paper afforded generation of a blue-colored PDA. The blue-colored filter paper was exposed to 10 mM of various amino acids in PBS buffer-EtOH mixture (1:1, v/v, pH: 7.4) for 30 min. Only cystein was found to induce a blue-to-red color change of the polymer.

Thermochromic Reversibility Test

A 1-endo immobilized filter paper was placed on a hot plate (110 ºC) for 5 min to induce the retro Diels-Alder reaction. The filter paper was annealed at either 35 ºC or 45 ºC for 18 h. UV irradiation (254 nm, 1 mW/cm^2, 1 min) to the annealed filter paper afforded generation of a blue-colored PDA. The blue-colored filter paper was subjected to thermal cycles (heating-cooling) and the color changes were monitored.

^{1}H, ^{13}C NMR and mass Spectra for F, M, 1-exo and 1-endo

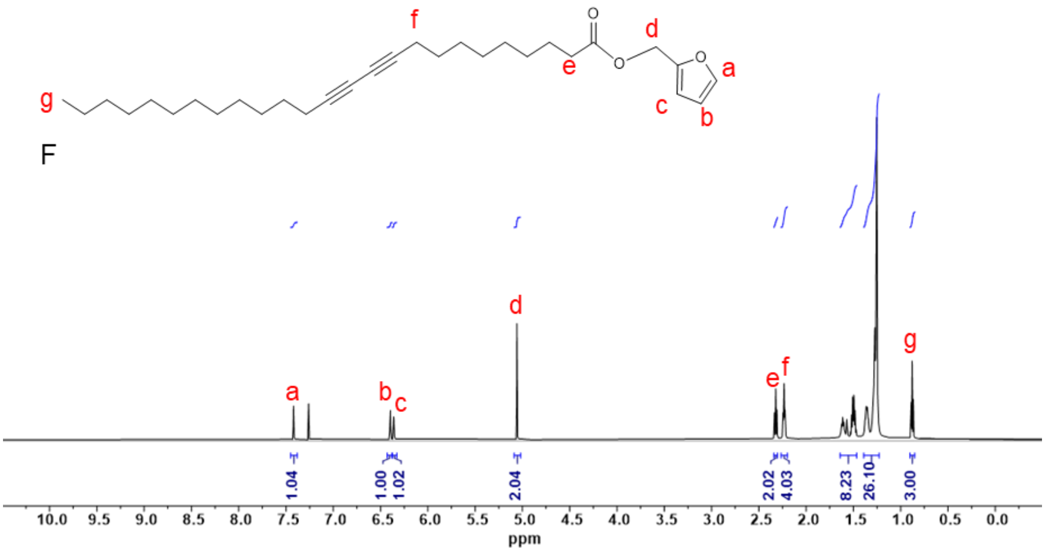

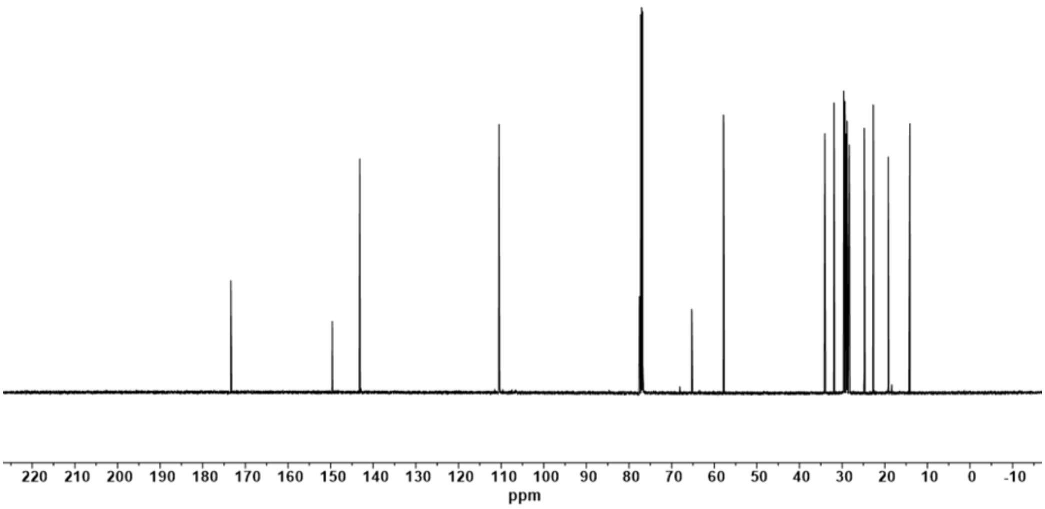

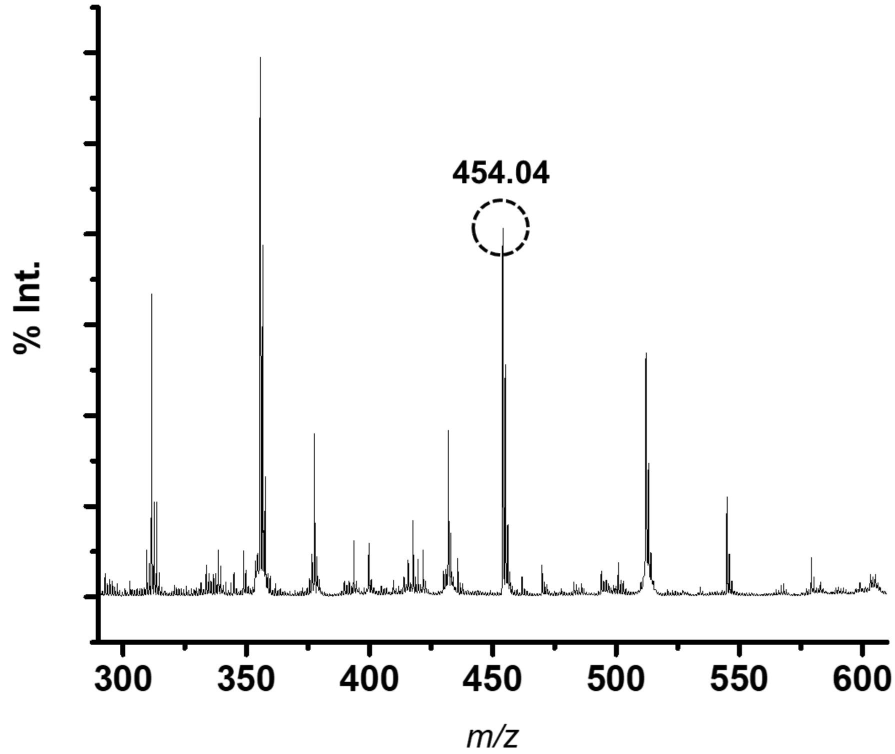

Supplementary Figure 3. ^{1}H (top, 600 MHz), ^{13}C (bottom, 150 MHz) NMR in $CDCl_3$ and MALDI-TOF spectra of F.

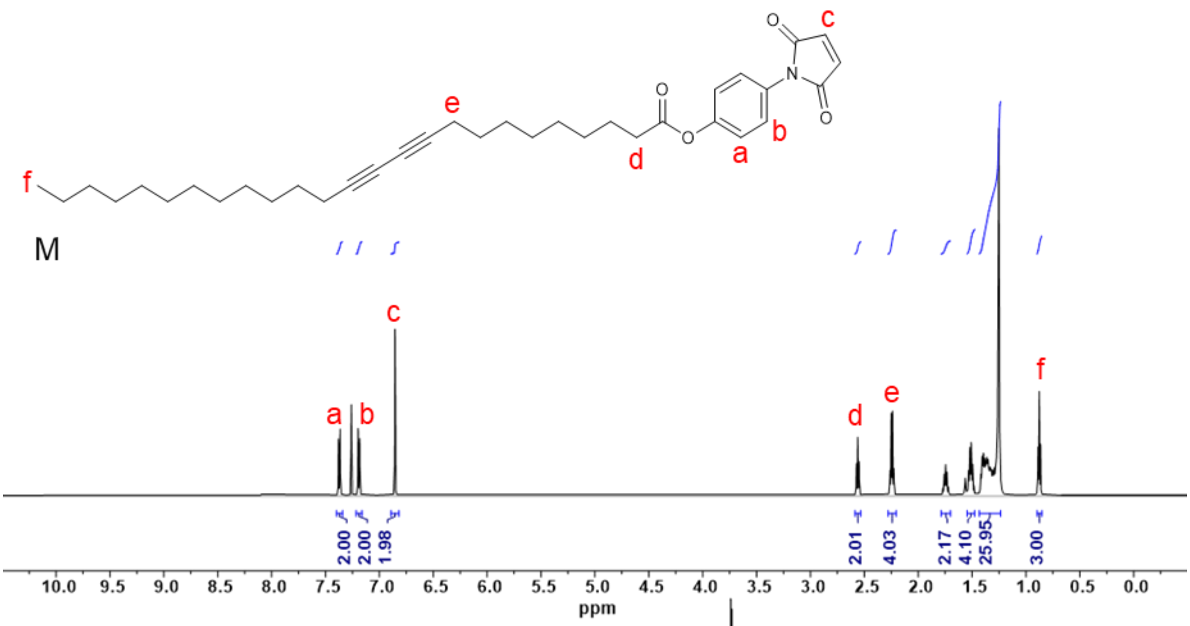
M
a
b
c
d
e
f
2.00
2.00
1.98
2.01
4.03
2.17
4.10
25.95
3.00
10.0 9.5 9.0 8.5 8.0 7.5 7.0 6.5 6.0 5.5 5.0 4.5 4.0 3.5 3.0 2.5 2.0 1.5 1.0 0.5 0.0
ppm

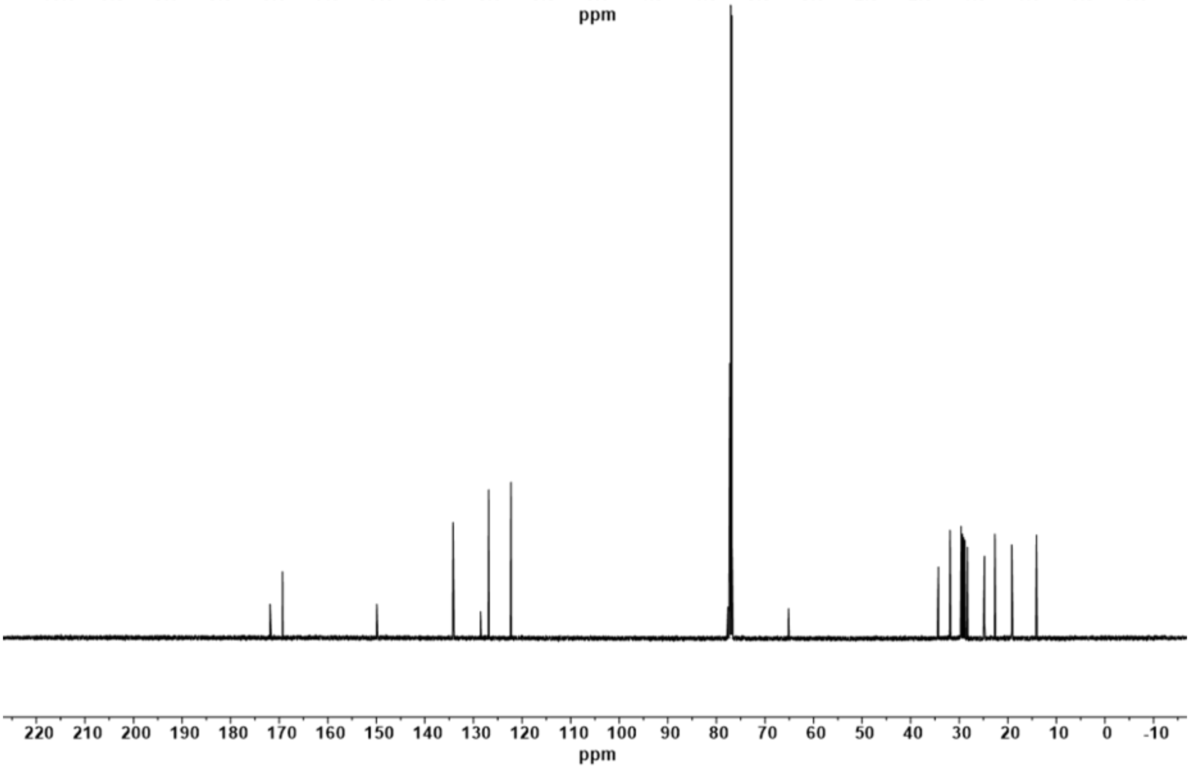
220 210 200 190 180 170 160 150 140 130 120 110 100 90 80 70 60 50 40 30 20 10 0 -10
ppm

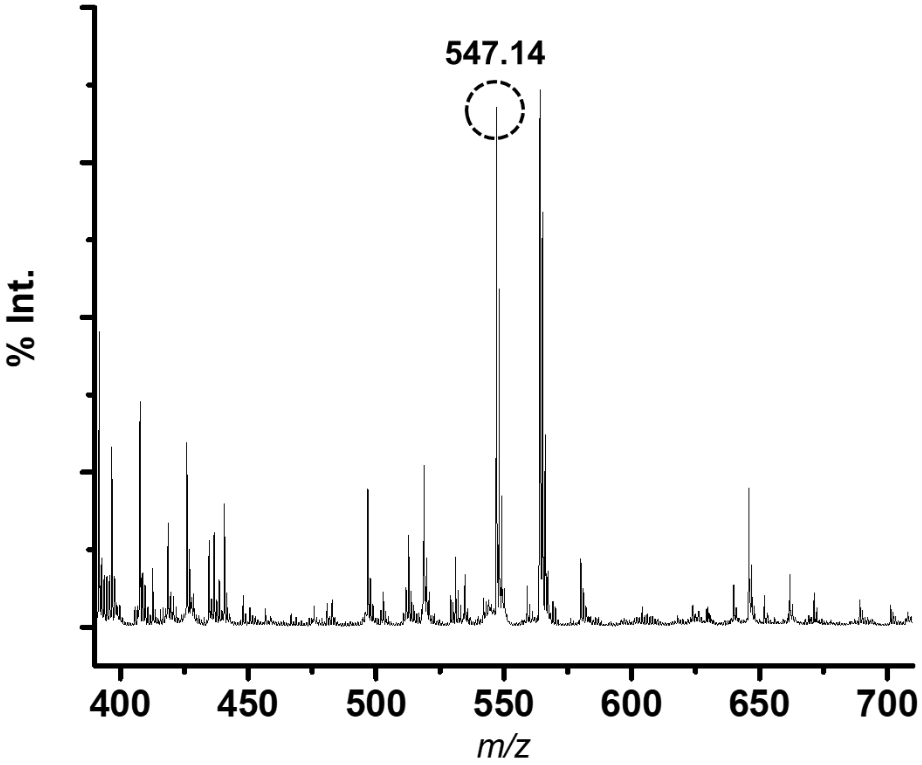

Supplementary Figure 4. ^{1}H (top, 600 MHz), ^{13}C (bottom, 150 MHz) NMR in $CDCl_3$ and MALDI-TOF spectra of M.

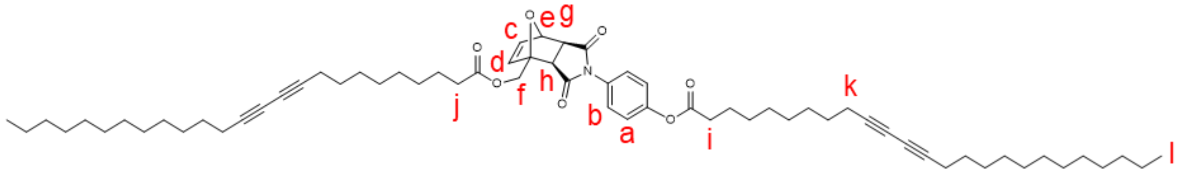
c
e
g
d
f
h
j
b
a
i
k
l

1-exo

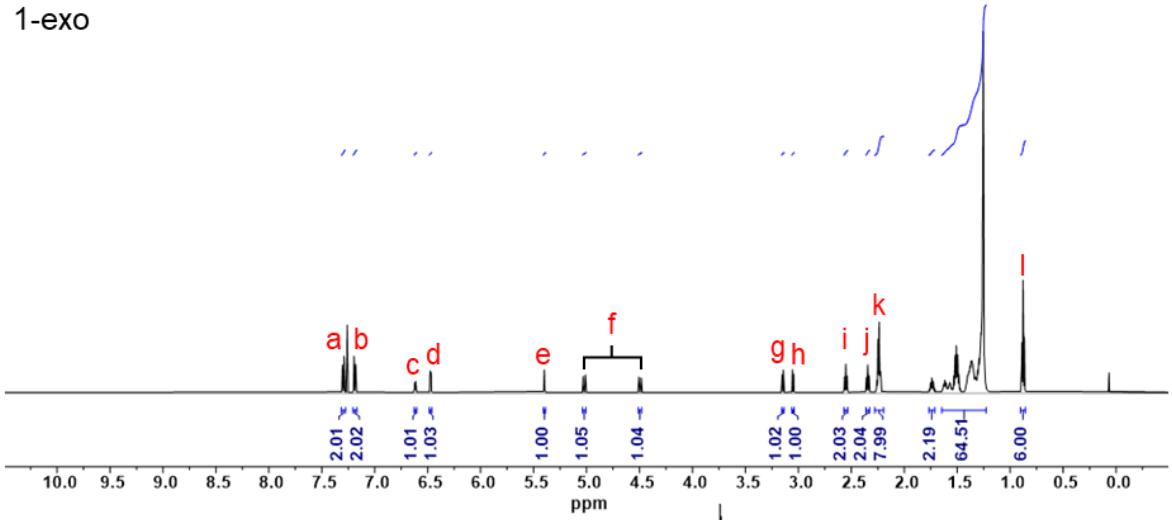
a
b
c
d
e
f
g
h
i
j
k
l
2.01
2.02
1.01
1.03
1.00
1.05
1.04
1.02
1.00
2.03
2.04
7.99
2.19
64.51
6.00
10.0 9.5 9.0 8.5 8.0 7.5 7.0 6.5 6.0 5.5 5.0 4.5 4.0 3.5 3.0 2.5 2.0 1.5 1.0 0.5 0.0
ppm

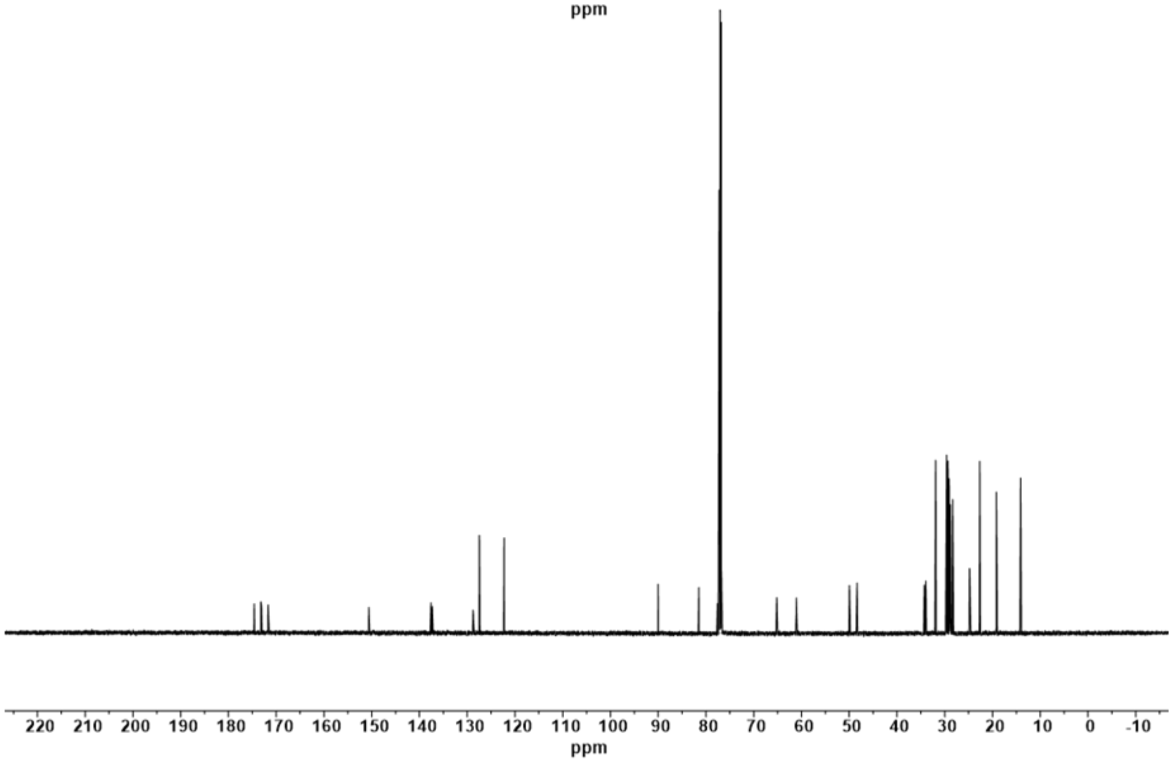
220 210 200 190 180 170 160 150 140 130 120 110 100 90 80 70 60 50 40 30 20 10 0 -10
ppm

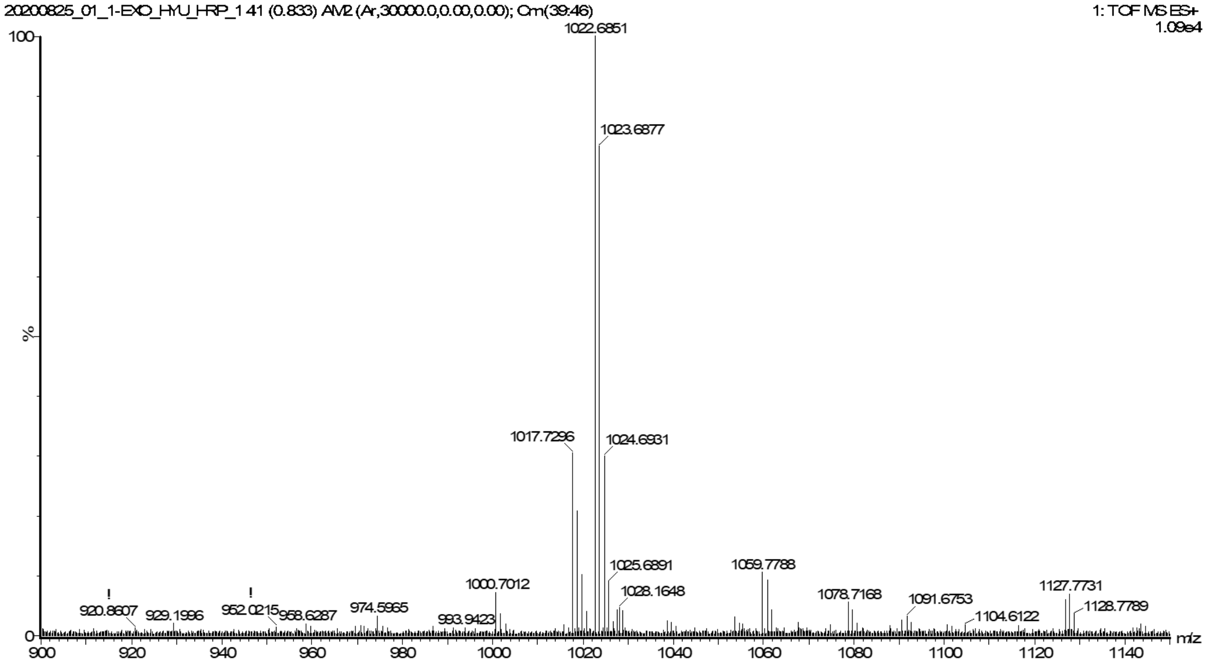

Supplementary Figure 5. ^{1}H (top, 600 MHz), ^{13}C (bottom, 150 MHz) NMR in $CDCl_3$ and High-resolution mass spectra of 1-exo

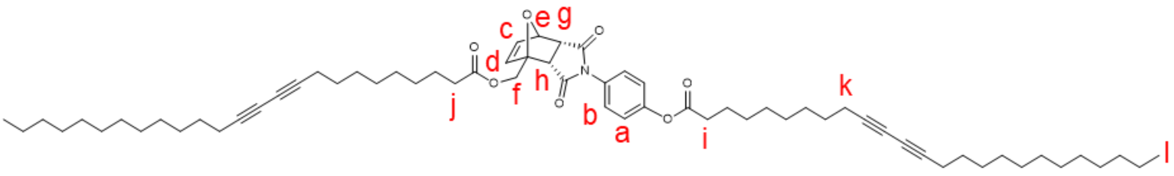
c
e
g
d
f
h
j
b
a
i
k
l

1-endo

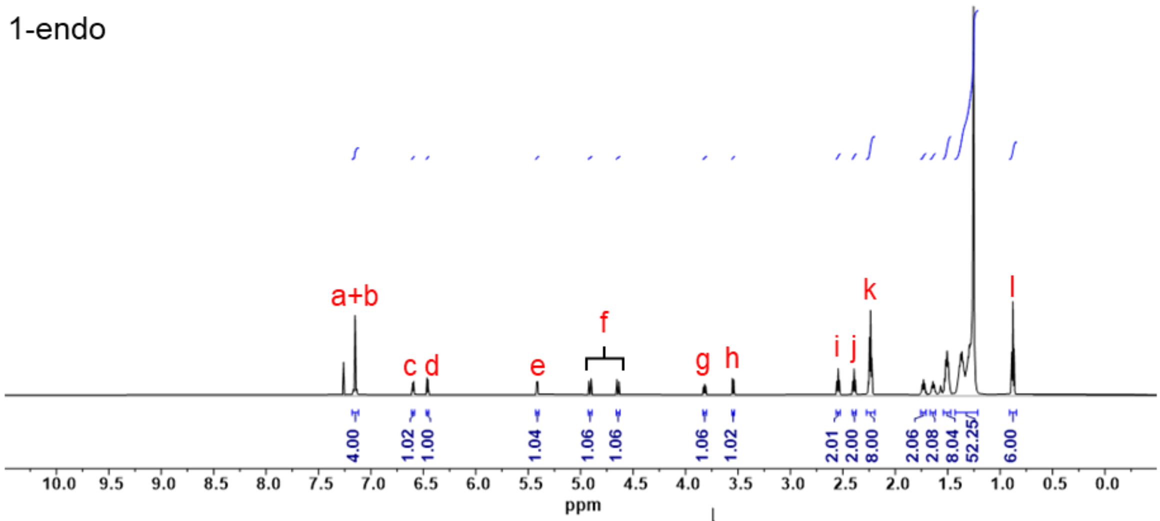
a+b
c d
e
f
g
h
i j
k
l
4.00
1.02
1.00
1.04
1.06
1.06
1.06
1.02
2.01
2.00
8.00
2.06
2.08
8.04
52.25
6.00
10.0 9.5 9.0 8.5 8.0 7.5 7.0 6.5 6.0 5.5 5.0 4.5 4.0 3.5 3.0 2.5 2.0 1.5 1.0 0.5 0.0
ppm

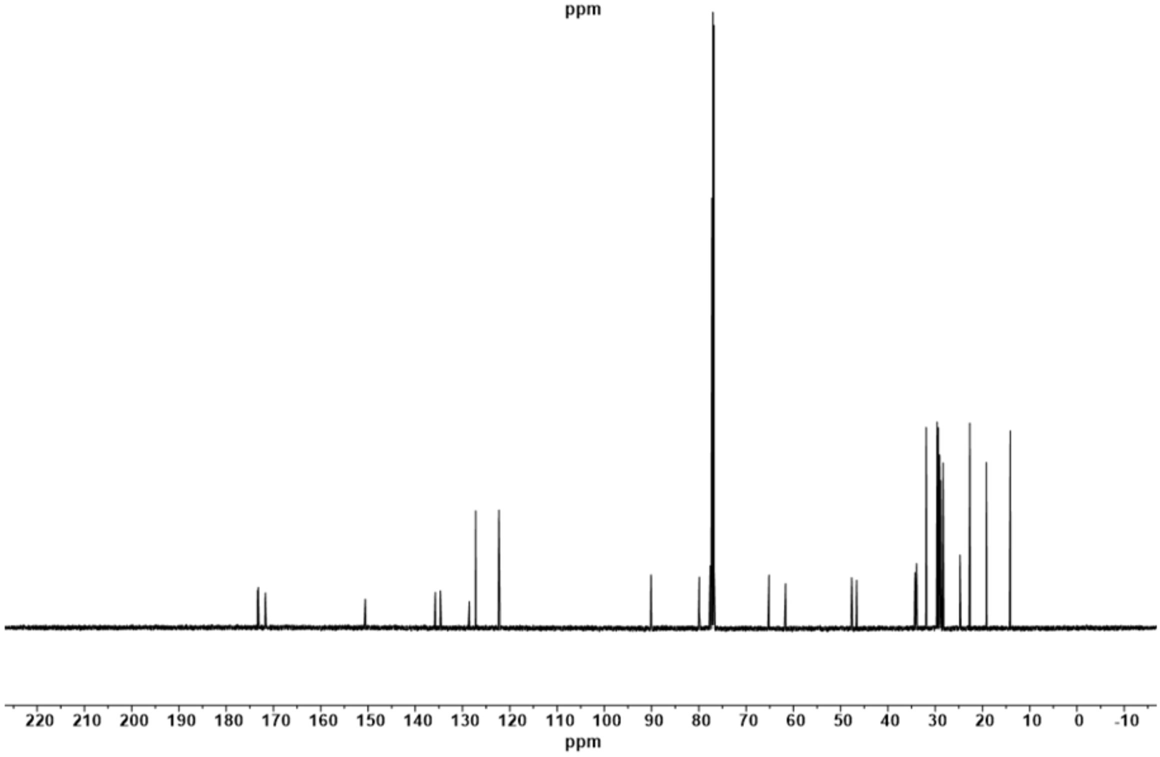
220 210 200 190 180 170 160 150 140 130 120 110 100 90 80 70 60 50 40 30 20 10 0 -10
ppm

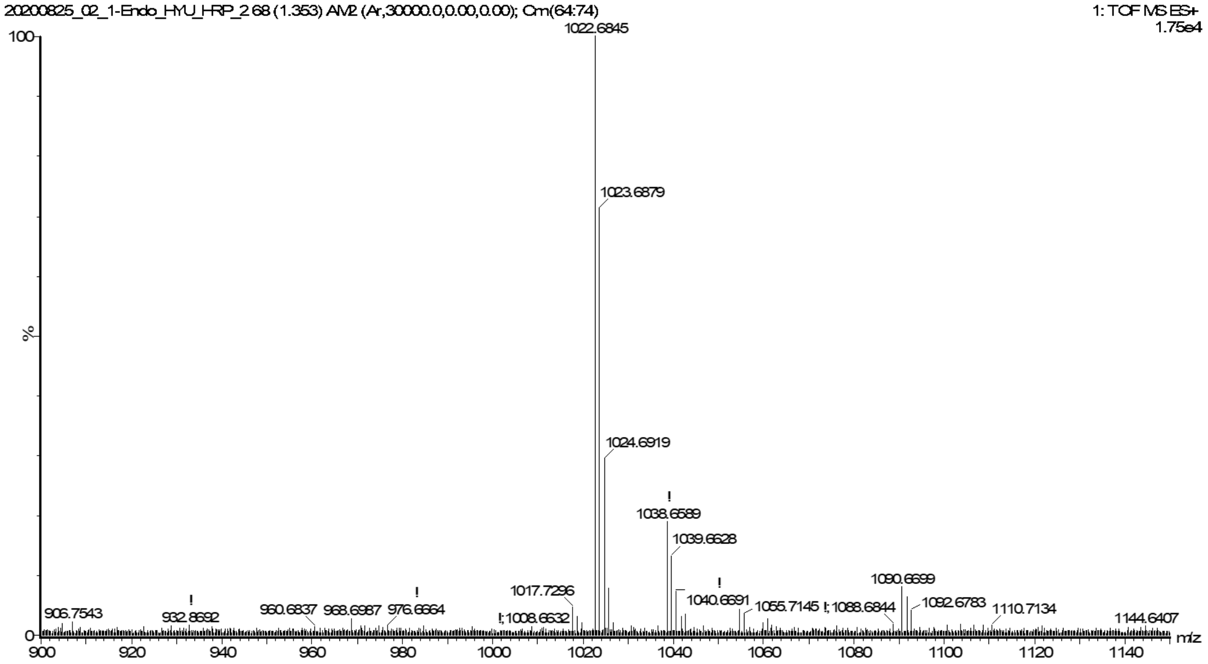

Supplementary Figure 6. ^{1}H (top, 600 MHz), ^{13}C (bottom, 150 MHz) NMR in $CDCl_3$ and High-resolution mass spectra of 1-endo

Supplementary Figures 7-25

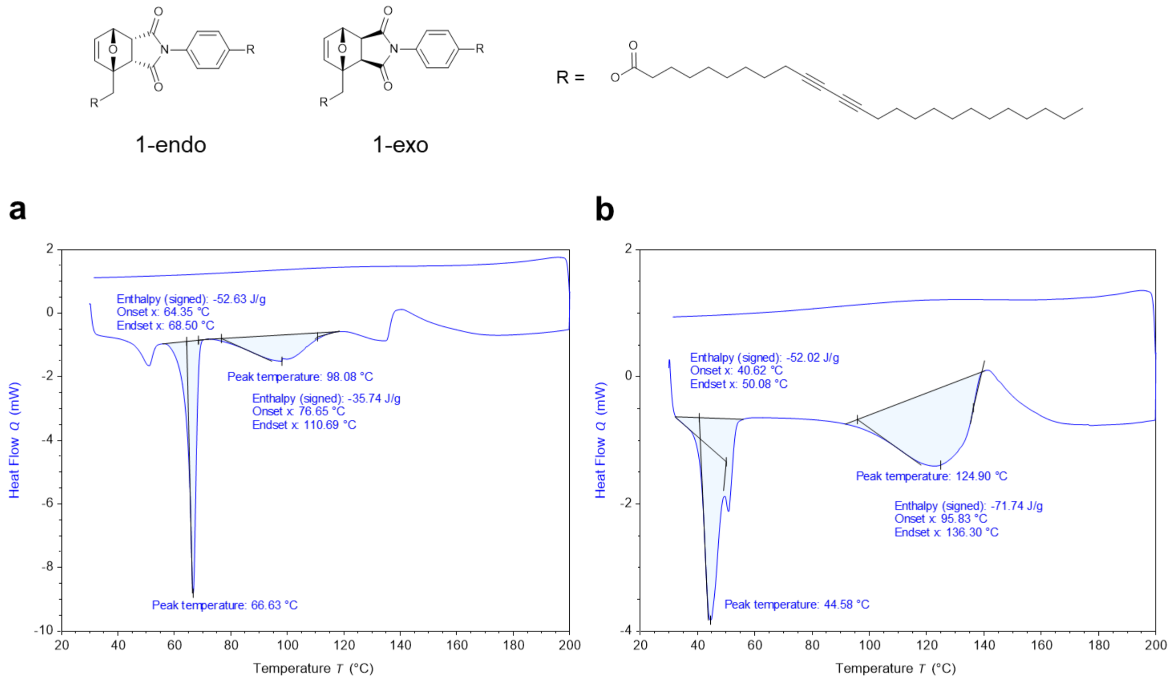

Supplementary Figure 7. Differential scanning calorimetry (DSC) thermograms of 1-endo (**a**) and 1-exo (**b**).

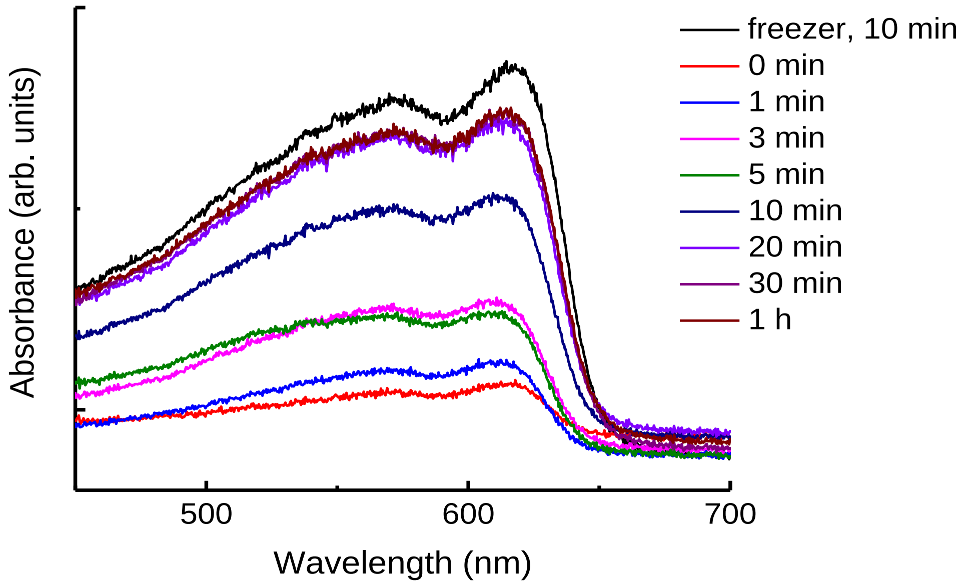

Supplementary Figure 8. UV-vis absorption spectra obtained after UV irradiation (254 nm, 1 mW/cm^2, 1 min) of 1-endo coated filter papers that were heated at 110 ºC for 5 min followed by keeping the papers at room temperature for designated times or in a freezer (-10 ºC) for 10 min.

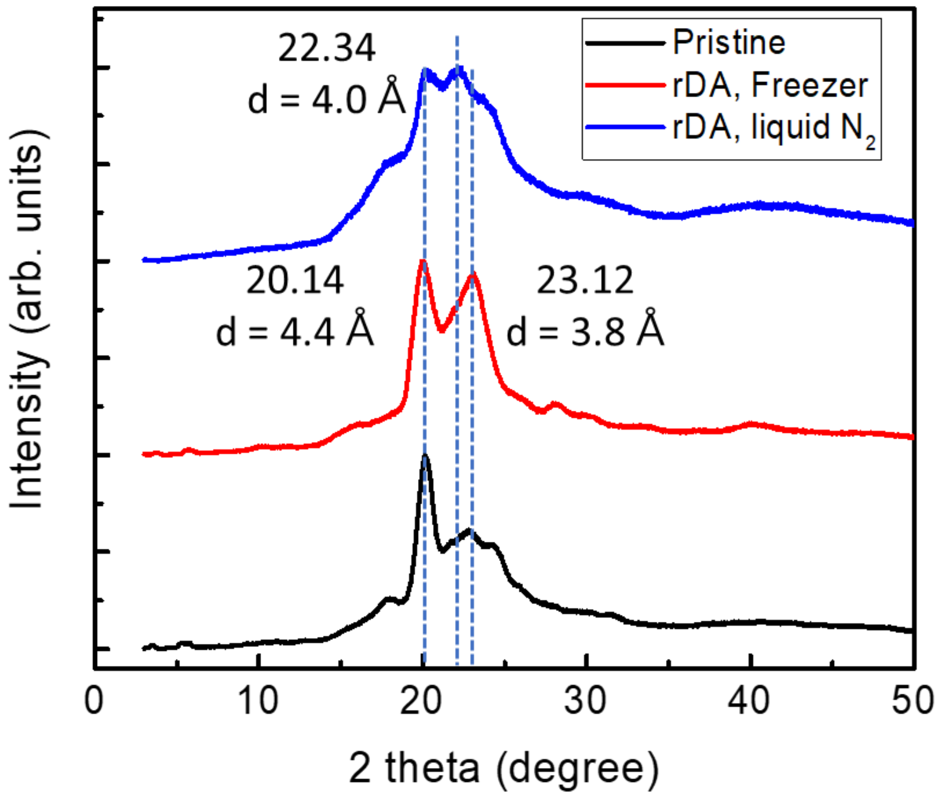

Supplementary Figure 9. Powder X-ray diffraction (XRD) spectra of 1-endo as prepared (black line) and obtained after heating of 1-endo at 110 °C for 1 min followed by 10 min in a freezer (red line) and after heating of 1-endo at 110 °C for 1 min immediately followed by quenching in a liquid nitrogen chamber (blue line).

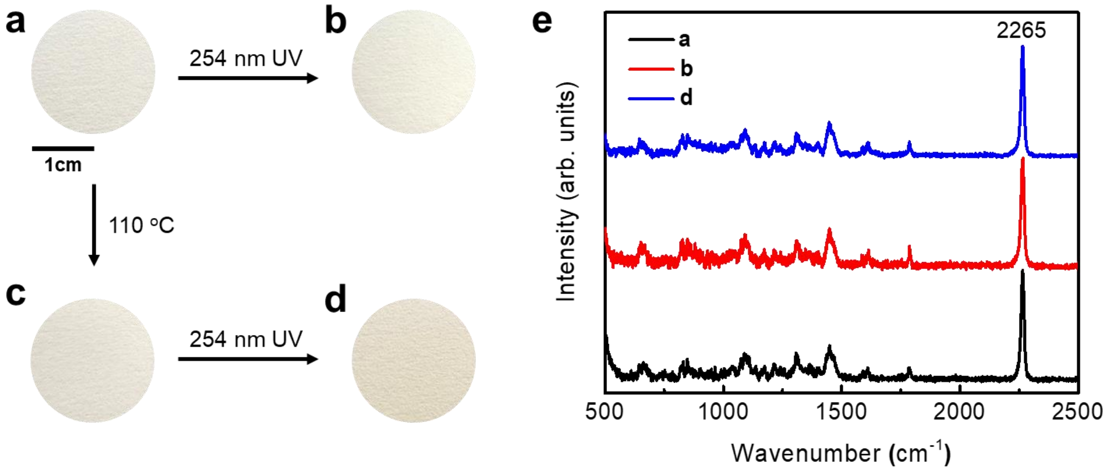

Supplementary Figure 10. a, Photographs of a 1-exo immobilized filter paper before (i) and after (ii) UV irradiation (254 nm, 1 mW/cm^2, 1 min). Photographs of the 1-exo immobilized filter paper obtained after heating the paper for 5 min at 110 °C (iii) followed by UV irradiation (254 nm, 1 mW/cm^2, 1 min) (iv). **b**, Raman spectra recorded with filter papers (i), (ii) and (iv) shown in **a**. Only unpolymerized monomeric diacetylene band at 2265 cm^{-1} is shown.

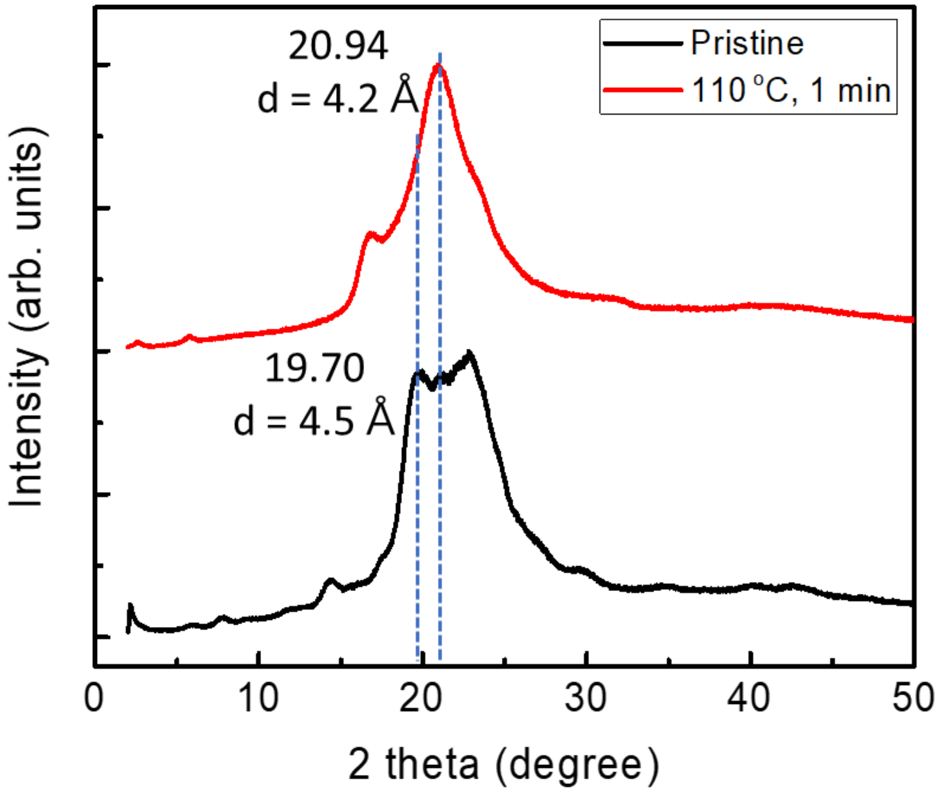

Supplementary Figure 11. Powder X-ray diffraction (XRD) spectra of 1-exo as prepared (black line) and after heating at 110 °C for 1 min (red line).

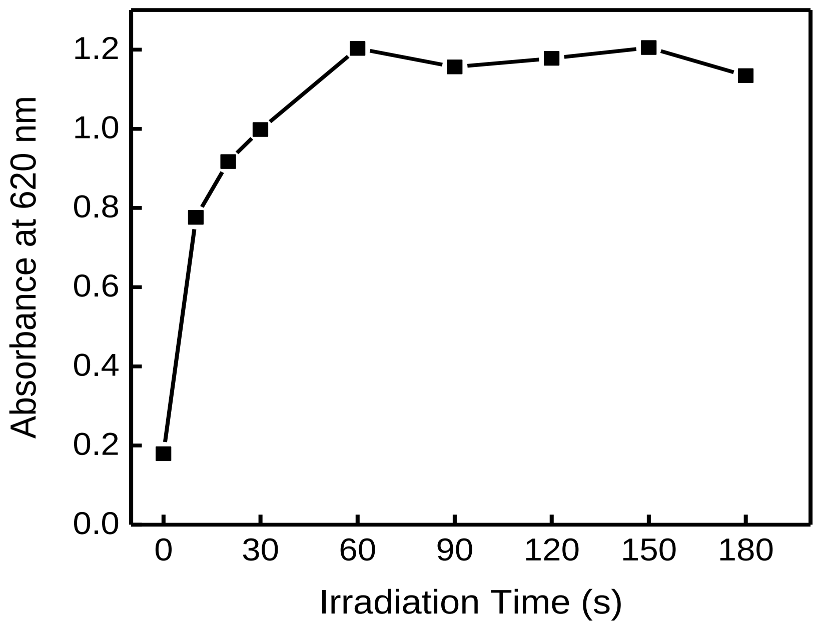

Supplementary Figure 12. Absorbance at 620 nm as a function of UV irradiation time of a 1-endo coated filter paper that was heated at 110 °C for 5 min followed by stabilization at -10 °C for 10 min.

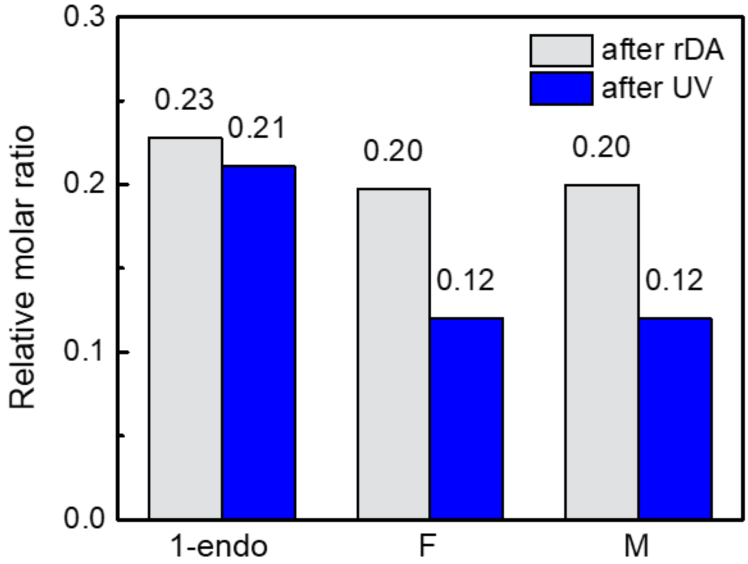

Supplementary Figure 13. Relative amount of residual compounds obtained after heat treatment (gray color) and UV irradiation (blue color) of 1-endo coated fileter paper. The gray colored bar graphs are obtained by heating 1-endo at 110 °C for 5 min and the blue colored bar graphs indicate the recovered 1-endo, F, and M obtained after UV induced polymerization (254 nm, 1 mW/cm^2, 1 min).

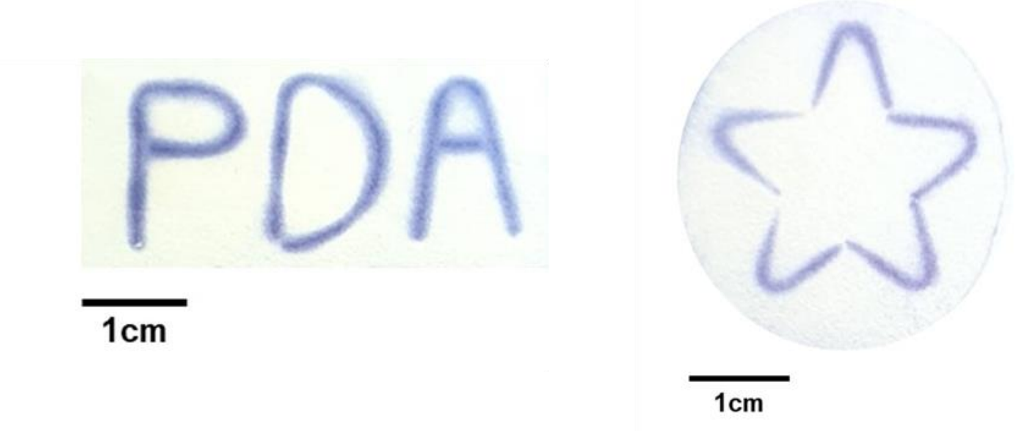

Supplementary Figure 14. Photographs of letter PDA (left) and star shape (right) images created by partial heat treatment and UV irradiation of 1-endo coated filter papers.

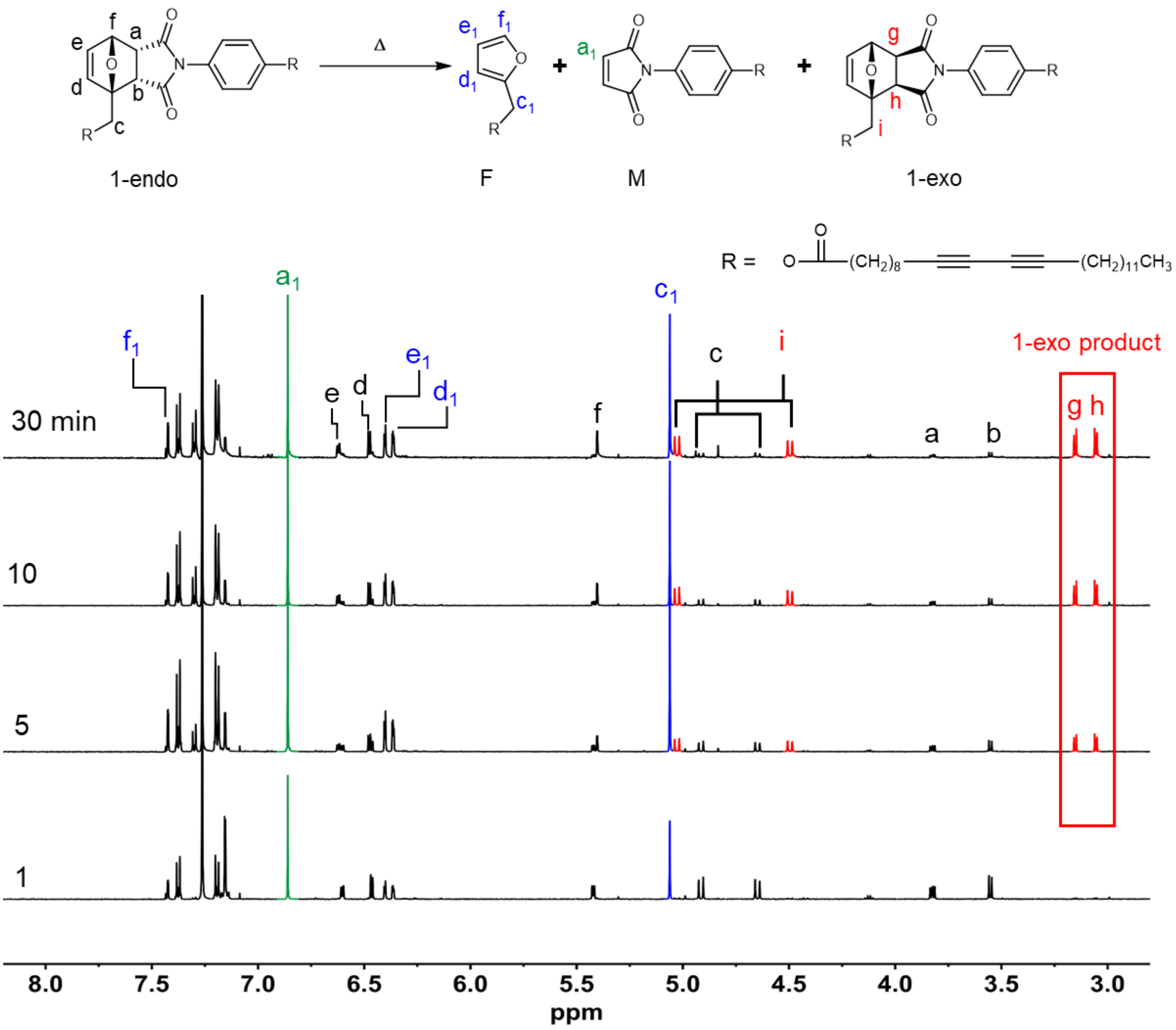

Supplementary Figure 15. ^{1}H NMR spectra (600 MHz, $CDCl_3$) obtained after heating of 1-endo on a glass substrate for various times at 110 °C.

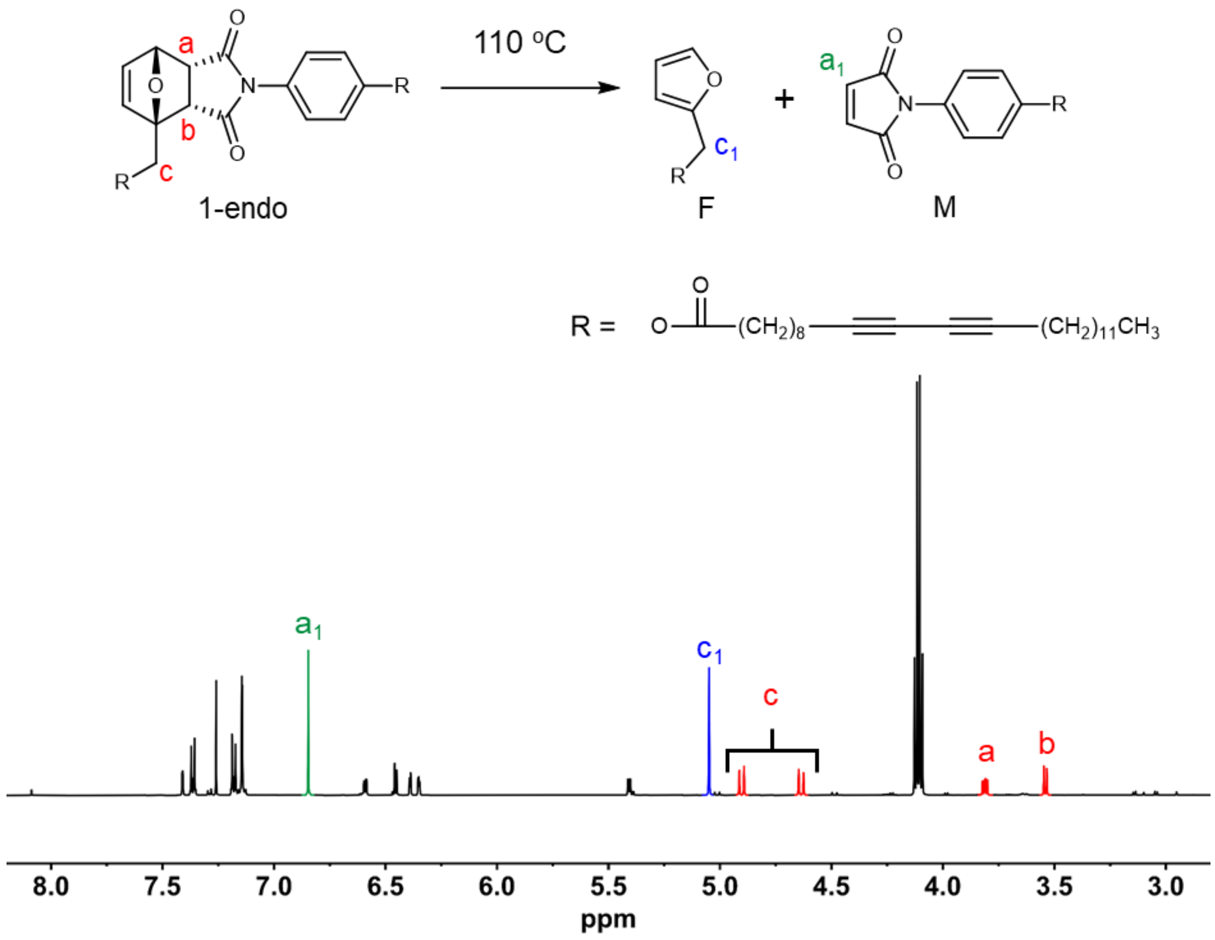

Supplementary Figure 16. ^{1}H NMR spectrum (600 MHz, $CDCl_3$) obtained after heat treatment of a 1-endo coated filter paper for 5 min at 110 °C.

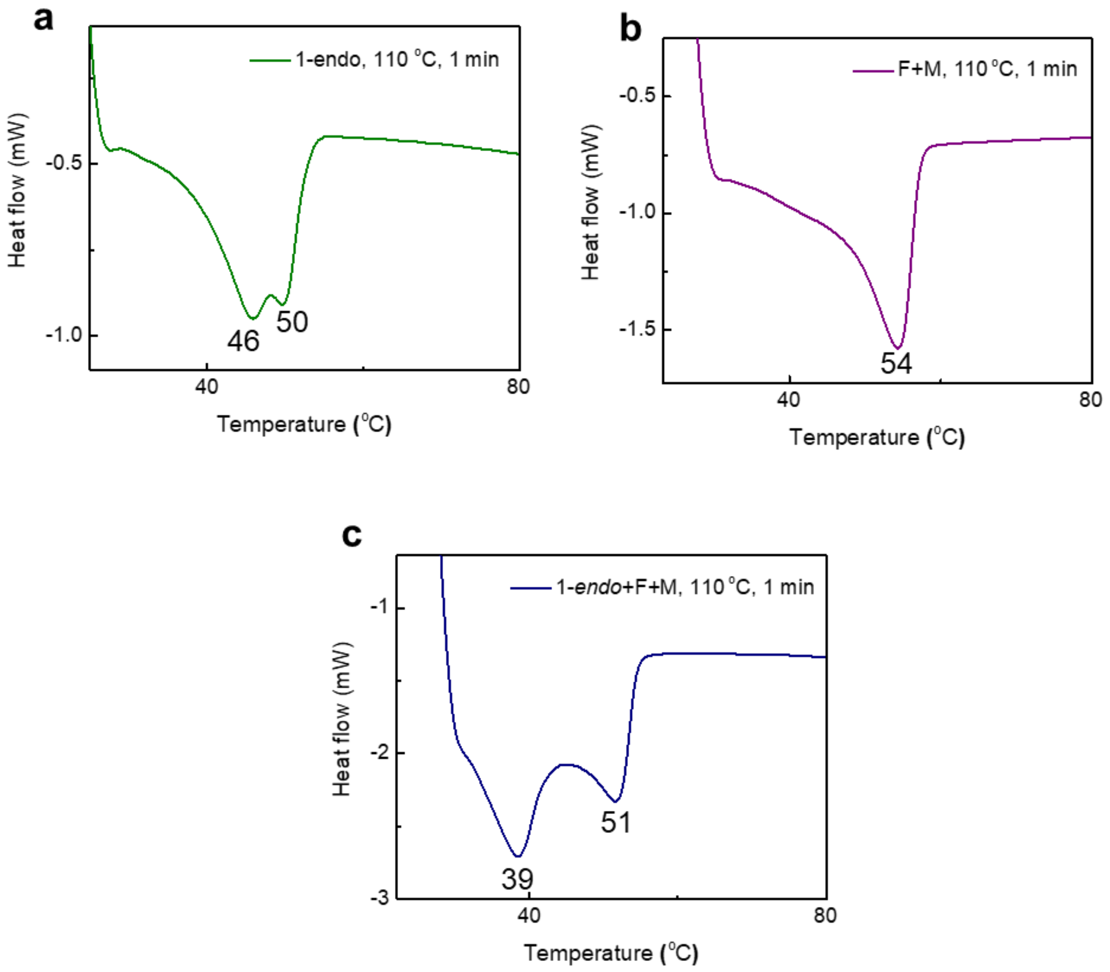

Supplementary Figure 17. a, Differential scanning calorimetry (DSC) thermogram of 1-endo obtained after heating at 110 °C for 1 min. **b**, DSC thermogram of a 1:1 mixture of F and M obtained after heating at 110 °C for 1 min. **c**, DSC thermogram of a 1:1:1 mixture of 1-endo, F and M obtained after heating at 110 °C for 1 min.

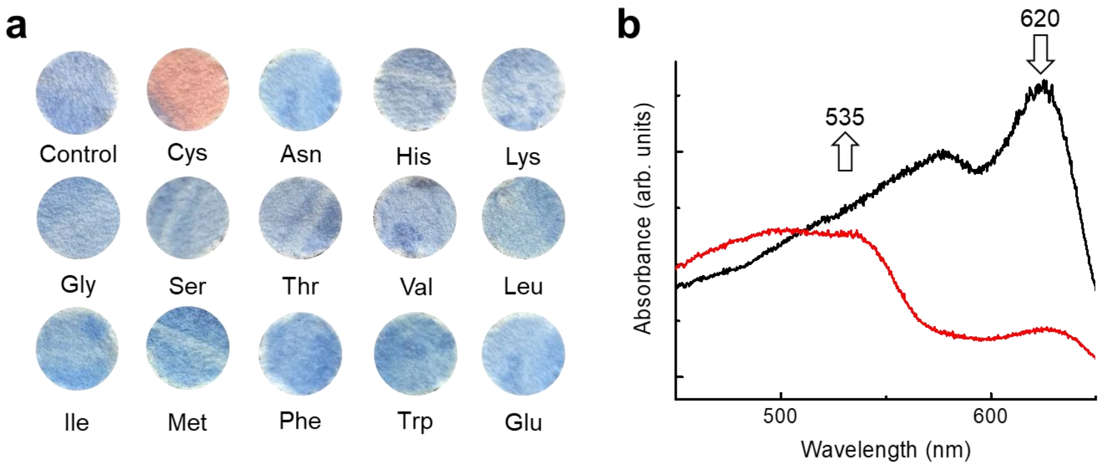

Supplementary Figure 18. a, Photographs of heat-treated (110 °C for 5 min) and UV irradiated (254 nm, 1 mW/cm^2, 1 min) M coated filter papers after exposure (30 min) to 10 mM of various amino acids in PBS buffer-EtOH mixture (1:1, v/v, pH: 7.4). **b**, Visible absorption spectra of a PDA coated filter paper (obtained as described in a) before (black line) and after (red line) exposure to 10 mM of cysteine.

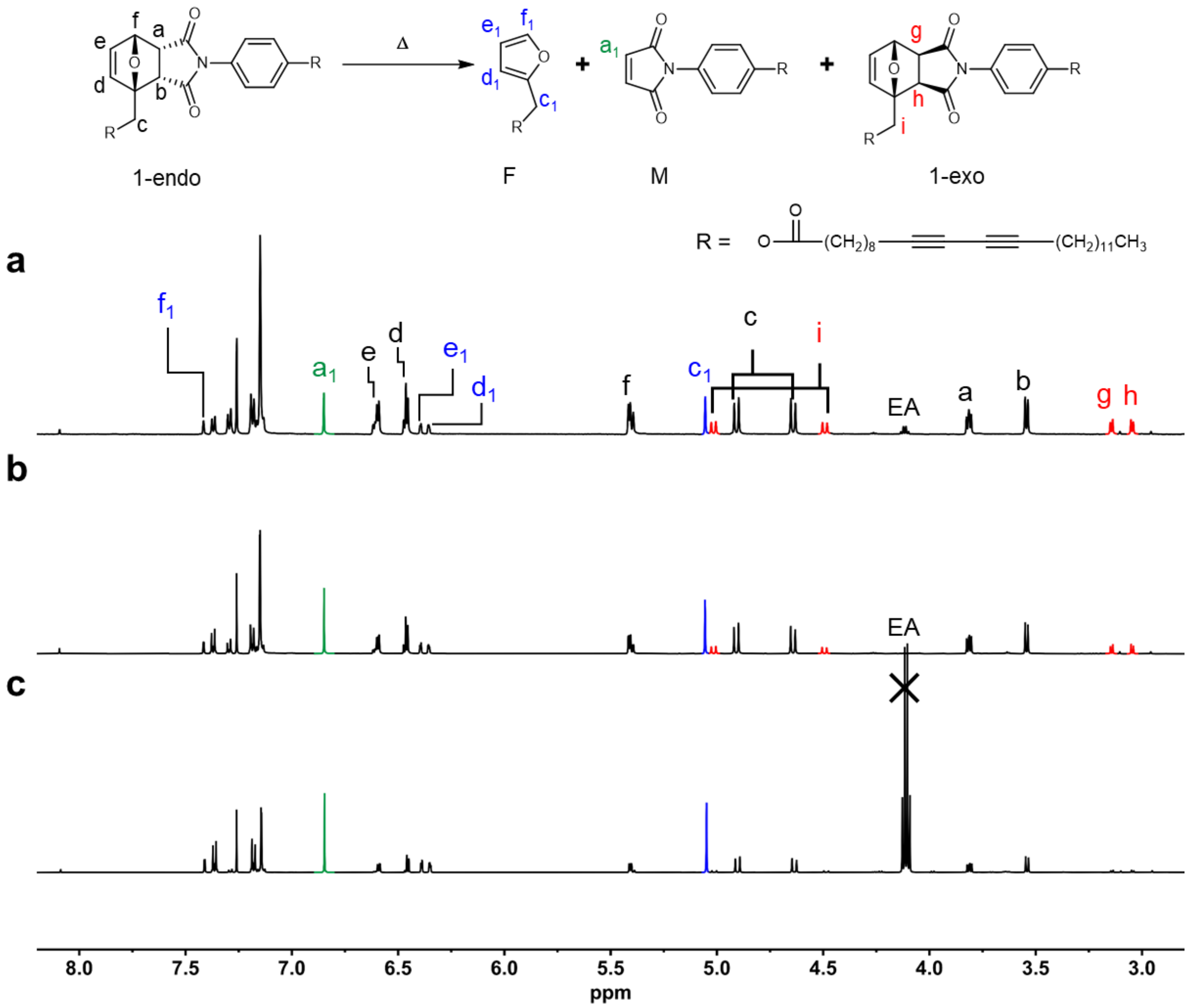

Supplementary Figure 19. ^{1}H NMR spectra of heat-treated (110 °C for 5 min) and UV irradiated (254 nm, 1 mW/cm^2, 1 min) 1-endo obtained without thermal annealing (**c**), after thermal annealing at 35 °C (**b**) and 45 °C (**a**) for 18 h, respectively.

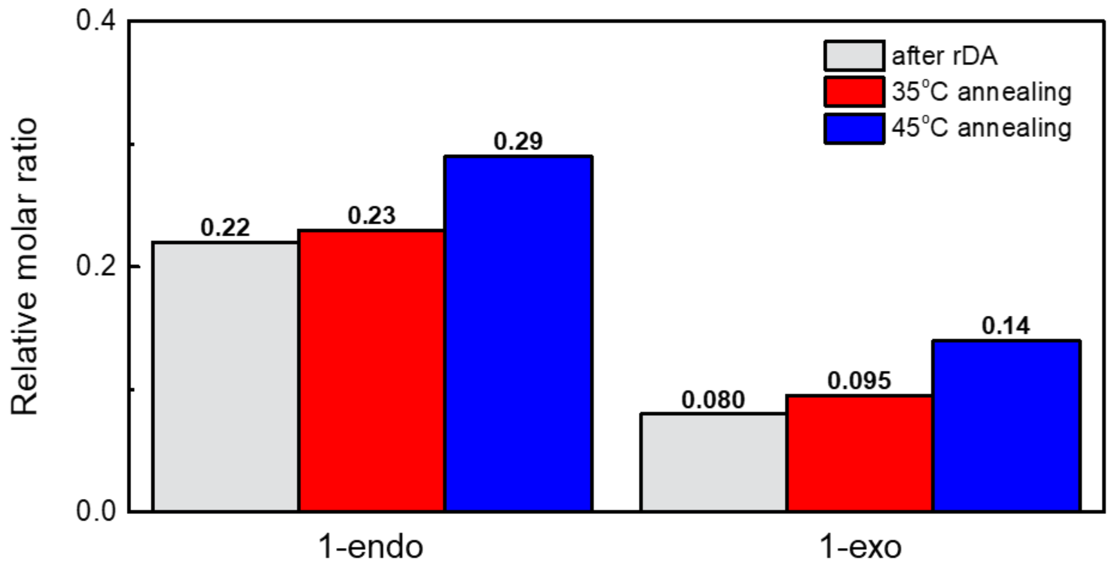

Supplementary Figure 20. Relative amounts of 1-endo and 1-exo obtained after retro Diels-Alder (rDA) (gray color) and after annealing the rDA sample at 35 ºC (red color) and 45 ºC (blue color) for 18 h. The data displayed in the figure were calculated based on the analysis of ^{1}H NMR spectra displayed in Supplemetary Fig. 19.

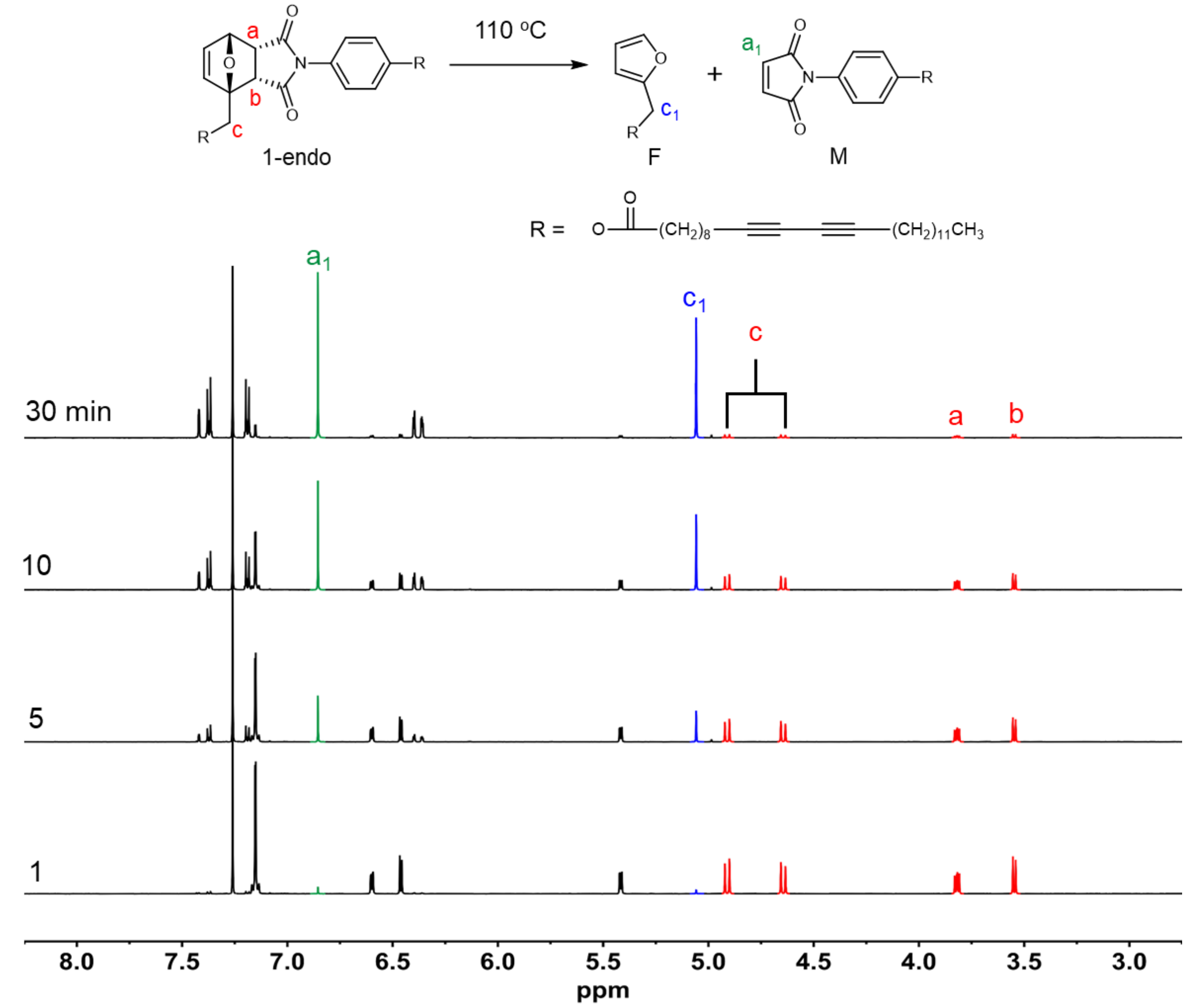

Supplementary Figure 21. ^{1}H NMR spectra (600 MHz) obtained after heating of 1-endo in $CDCl_3$ for various times at 110 °C. In order to prevent solvent evaporation during the heating process, the NMR tube was sealed tightly with teflon tape and paraffin film.

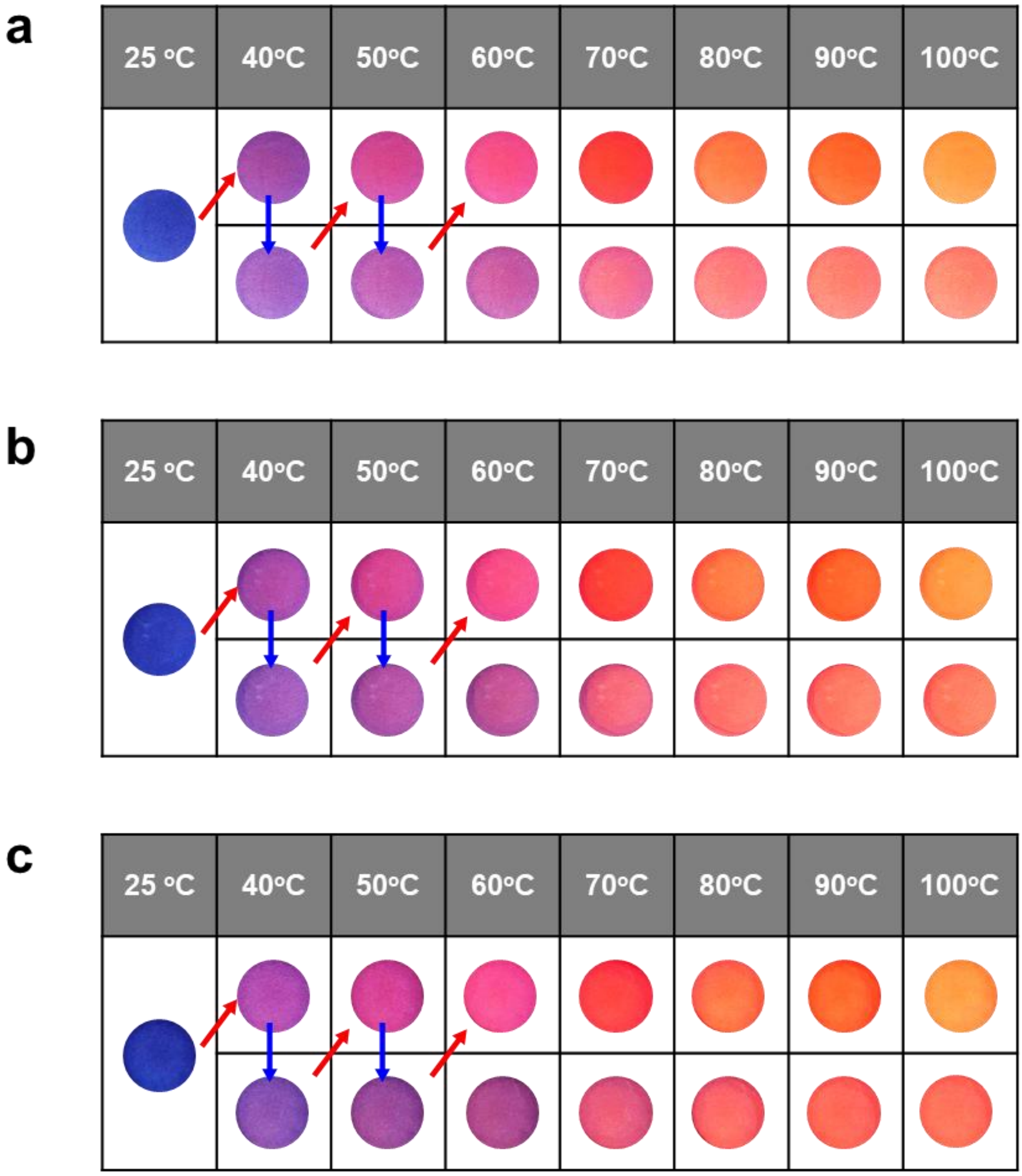

Supplementary Figure 22. a-c, Photographs of heat-treated (110 °C, 30 min in $CDCl_3$), self-assembled and UV irradiated (254 nm, 1 mW/cm^2, 1 min) 1-endo coated filter papers upon thermal cycling.

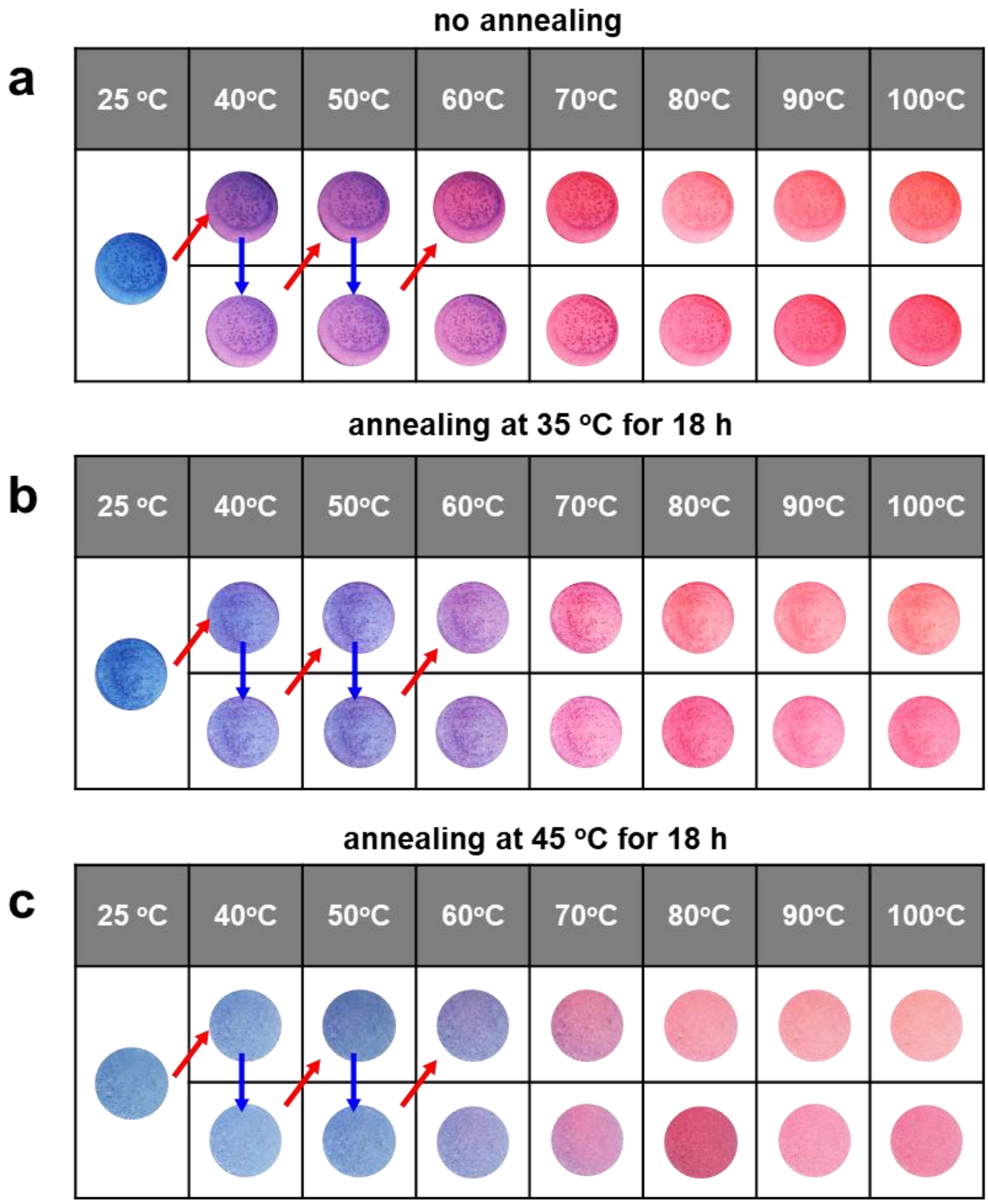

Supplementary Figure 23. a-c, Photographs of self-assembled and UV irradiated (254 nm, 1 mW/cm^2, 1 min) filter papers coated with 1:1 mixture of F and M upon thermal cycling. Polymerization was conducted without thermal annealing (**a**), after thermal annealing at 35 °C (**b**) and 45 °C (**c**) for 18 h, respectively.

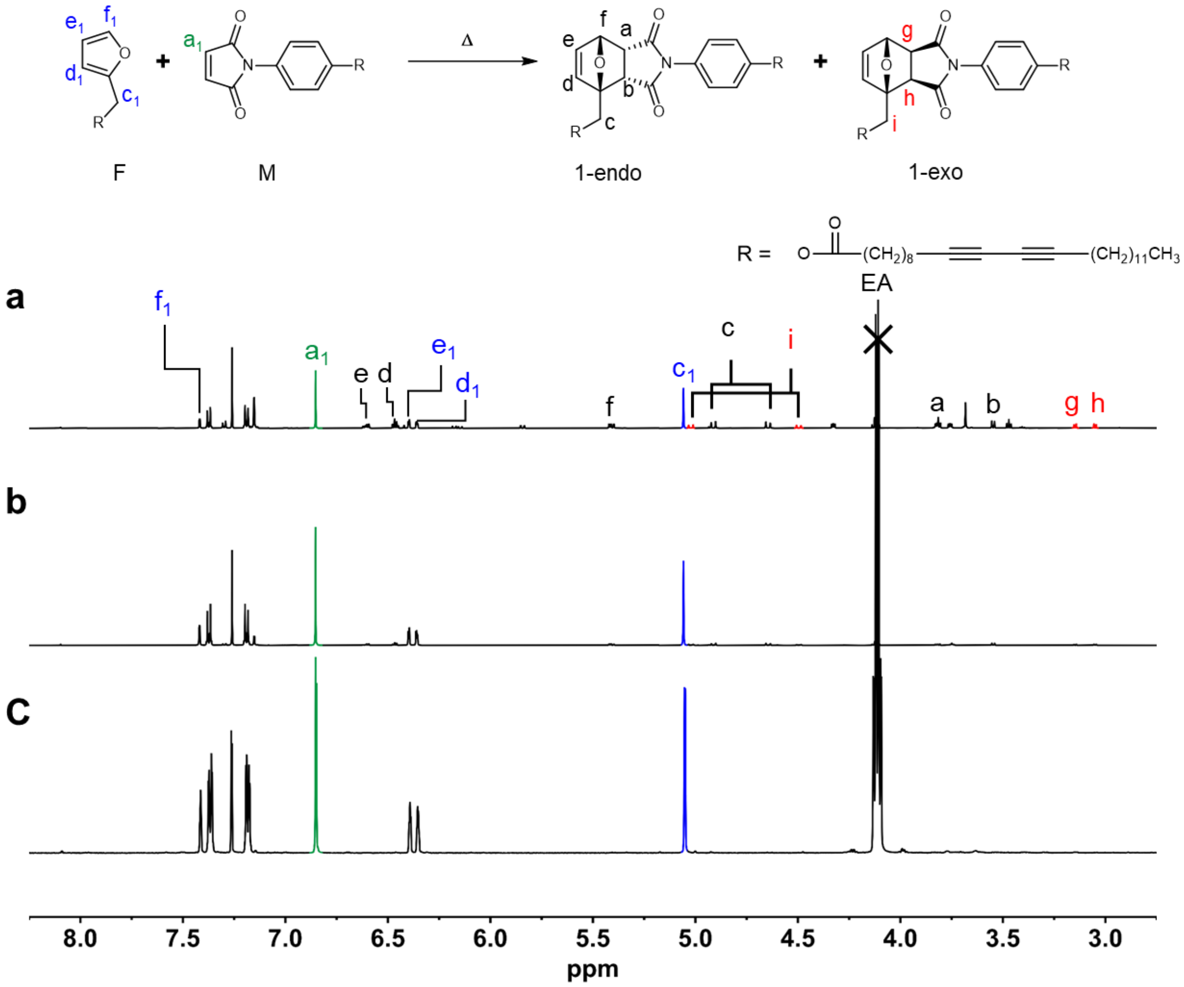

Supplementary Figure 24. ^{1}H NMR spectra of self-assembled and UV irradiated (254 nm, 1 mW/cm^2, 1 min) F and M (1: 1 mixture) obtained without thermal annealing (**c**), after thermal annealing at 35 °C (**b**) and 45 °C (**a**) for 18 h, respectively.

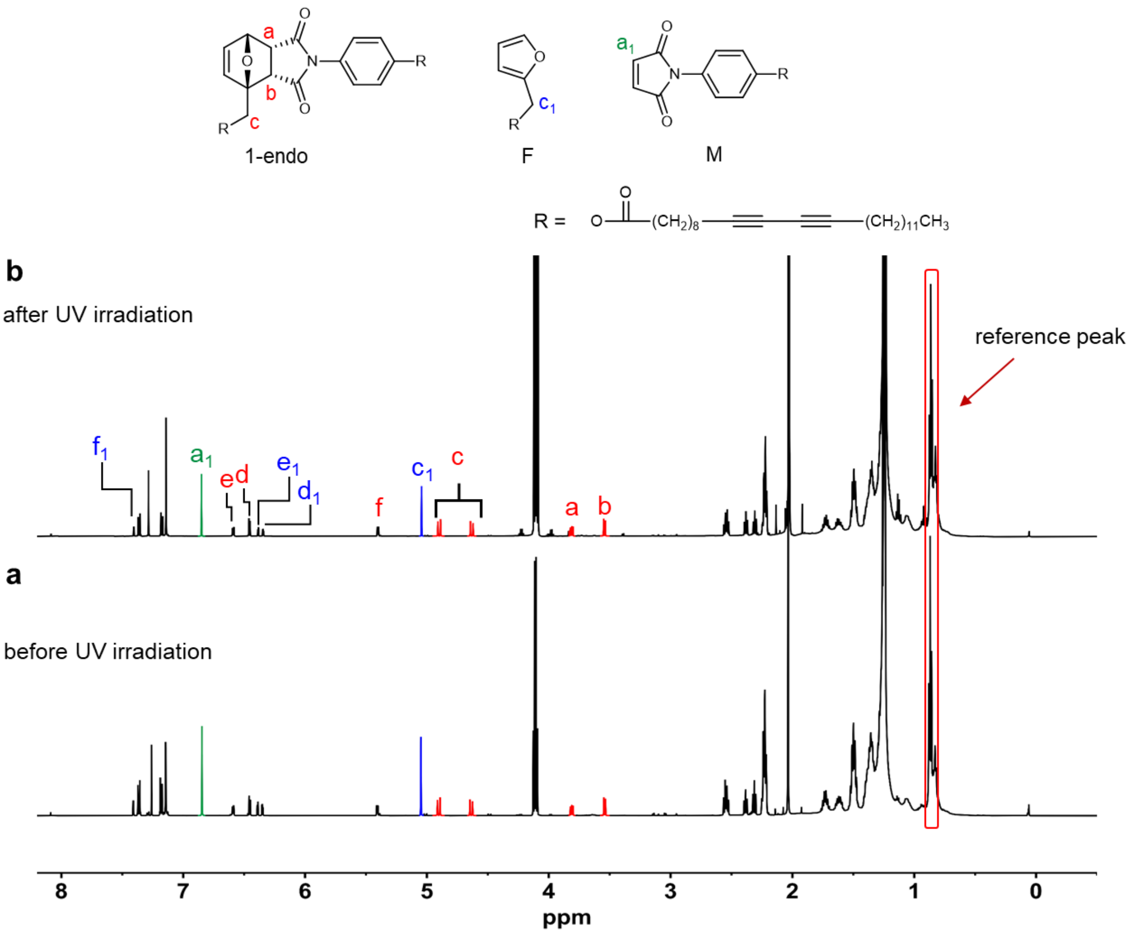

Supplementary Figure 25. ^{1}H NMR spectra (600 MHz, $CDCl_3$) obtained before (a) and after (b) UV irradiation (254 nm, 1 mW/cm^2, 1 min) of a 1-endo coated filter paper. The filter paper was subjceted to heating for 5 min at 110 °C before UV irradiation. The filter paper was washed with ethyl acetate to remove insoluble polymer and the ethyl acetate was evaporated under vaccum. The residue was dissolved in $CDCl_3$ for ^{1}H NMR analysis.

Supplementary Table 1

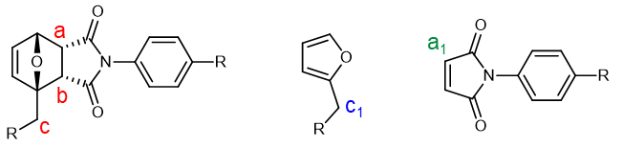

Sample	1-endo						F				M
	a	b	c	d	e	f	c_1	d_1	e_1	f_1	a_1
before	0.23	0.23	0.46	0.24	0.22	0.22	0.50	0.19	0.18	0.17	0.40
after UV	0.26	0.21	0.44	0.20	0.20	0.20	0.30	0.11	0.11	0.11	0.24

Supplementary Table 1. Relative integration values obtained before and after UV irradiation.

2. Supplementary References

1. Taimoory, S. M., Sadraei, S. I., Fayoumi, R. A., Nasri, S., Revington, M., Trant, J. F., Preparation and Characterization of a Small Library of Thermally-Labile End-Caps for Variable-Temperature Triggering of Self-Immolative Polymers. *J. Org. Chem.* **83**, 4427-4440 (**2018**).
